# Patient-specific modeling identifies metabolic interventions for reversing glucose use reprogramming in alcohol-associated hepatitis

**DOI:** 10.1038/s42003-026-10407-5

**Published:** 2026-06-04

**Authors:** Alexandra Manchel, Radhakrishnan Mahadevan, Jan B. Hoek, Ramon Bataller, Rajanikanth Vadigepalli

**Affiliations:** 1 Daniel Baugh Institute for Functional Genomics and Computational Biology, Department of Pathology and Genomic Medicine, Thomas Jefferson University, Philadelphia, PA USA; 2 Department of Chemical Engineering and Applied Chemistry, University of Toronto, Toronto, ON Canada; 3 The Institute of Biomedical Engineering, University of Toronto, Toronto, ON Canada; 4 The Liver Unit, Hospital Clinic, Barcelona, Spain; 5 Division of Epidemiology, Biostatistics, and Preventive Medicine, Department of Internal Medicine, School of Medicine, University of New Mexico Health Sciences Center, Albuquerque, NM USA

**Keywords:** Systems biology, Cell biology

## Abstract

Alcoholic hepatitis (AH) is an acute form of alcohol-associated liver disease with very few treatment options. Recent studies highlighted liver metabolic reprogramming in AH as an indicator of severity. We aim at identifying new intervention points to reverse liver metabolic dysregulation across varying degrees of AH. We develop 89 personalized genome-scale metabolic models by integrating a generic human cellular metabolic model with liver transcriptomics data from AH patients with varying disease severity and healthy controls. We grade the AH patients based on the model-predicted level of glycolysis reprogramming and validate the results using published metabolomics data. We test in silico gene knockdown interventions to reverse the aberrant metabolic reprogramming in AH. Knockdown of two glycolytic genes, *Hkdc1* and *Pkm*, significantly rebalance the metabolic fluxes toward a healthy liver metabolic phenotype. We use machine learning on the glycolysis fluxes to develop a quantitative glucose use reprogramming score, which correlates with AH severity and patient-specific responses to in silico gene knockdown interventions. The score was independently validated using a published AH liver transcriptomics dataset. We propose a cellular metabolism-based therapy targeting *Hkdc1* and *Pkm* in the glycolysis pathway as a potential treatment for reversing the aberrant glucose metabolism in AH.

## Abstract (web summary)

Patient-specific metabolic modeling of alcohol-associated hepatitis points to HKDC1 and PKM as key glycolytic pathway enzymes driving the deregulation of glucose use underlying pathological metabolic reprogramming

## Introduction

Alcohol-associated liver disease (ALD), which may result from excessive alcohol intake leading to significant liver damage, contributes greatly to global morbidity and mortality^1,2^. Alcohol-associated hepatitis (AH) is an acute form of ALD, which is characterized by hepatic inflammation, a rapid onset of jaundice, portal hypertension, and other liver-related complications^3^. Currently, the only pharmacological treatment is corticosteroids, which increase the risk of severe infections and have been shown to improve survival in only a subset of patients^3,4^. However, abstinence from alcohol remains the only effective factor contributing to long-term survival^3,5^. Therefore, there is a critical need for therapeutic advancements for patients with AH.

The liver plays a crucial role in metabolism and is specifically responsible for the oxidation of ethanol (alcohol) to acetaldehyde. When excessive alcohol is consumed, in the case of AH, reactive oxygen species (ROS) may be generated, leading to the AH phenotype of lipid accumulation, inflammation, and fibrosis^6,7^. Because the liver is responsible for many metabolic functions, it is also of interest to consider how other metabolic pathways may be dysregulated in patients at various stages of AH (early to severe) and how they compare to patients with non-alcohol-associated liver diseases (NALD). Recent studies have revealed that there is significant metabolic reprogramming in the livers of patients with severe AH^8,9^, which has fueled the growing interest in developing cell metabolism-based therapies^10^. In one such study, liver transcriptomics and metabolomics data were collected from patients with AH and healthy controls and were collectively analyzed to identify glucose metabolism as a major metabolic pathway with significant reprogramming in patients with AH as compared to healthy controls^8^. Among the many differentially expressed genes, HKDC1 was found to be most upregulated in patients with AH vs. healthy controls^8^. Further experimentation in primary rat hepatocytes identified the increased HKDC1 activity as a major contributor to the accumulation of glucose-6-phosphate (G6P) and glycogen^8^. In an additional study where transcriptomics data was collected from livers of patients with AH, major metabolic pathways contributing to basic hepatocellular function, including the metabolism of amino acids and lipids, biological oxidations, mitochondrial function, and bile acid metabolism, were significantly downregulated^9^.

We sought to study the entire metabolic landscape of the livers of patients with AH. Therefore, we utilized genome-scale metabolic modeling (GEM) methodologies to capture a systems-level understanding of the metabolic reprogramming occurring in the livers of patients with AH. A multitude of previous studies have developed GEMs to better understand the underlying metabolic mechanisms of the liver^11–20^. In a previous study from our group, disease state-specific GEMs were generated by integrating a generic human cellular metabolic model with liver RNA-seq data corresponding to multiple liver disease states, thereby providing unique insight into the metabolic pathways whose predicted metabolic fluxes activity significantly differed in AH vs. NALD vs. healthy patients^20^. We found there to be sequential dysregulation of the solute transport mechanisms underlying the glutathione metabolic pathway with increasing AH disease severity, which is not observed with NALD^20^. While that study was the first of its kind to generate personalized GEMs specific to various AH states, we were unable to compare the metabolic activity between patients within and across a disease state at the patient-specific level.

Therefore, in this study, we integrate liver transcriptomics data with a generic GEM to study the metabolic flux activity of individual patients at progressing stages of AH to uncover specific locus points of metabolic reprogramming. Further, we compare the predicted metabolic fluxes and the associated gene expression of the encoded enzymes for each reaction within a metabolic subsystem to determine which genes may be driving the system’s metabolic activity. We then perform a series of in silico gene knockdown experiments to determine if the metabolic fluxes of patients with severe ALD can be rebalanced towards a healthy phenotype. Finally, we develop a machine learning regression model that can accurately capture the extent of metabolic reprogramming and predict patient responses to in silico gene knockdown interventions. Thus, we have identified specific points of metabolic reprogramming in AH patients that can drive the development of cellular metabolism-based therapeutics for alternative and novel treatment of ALD.

## Results

### Metabolic gene expression informs patient-specific genome-scale metabolic models

**Fig. 1:**
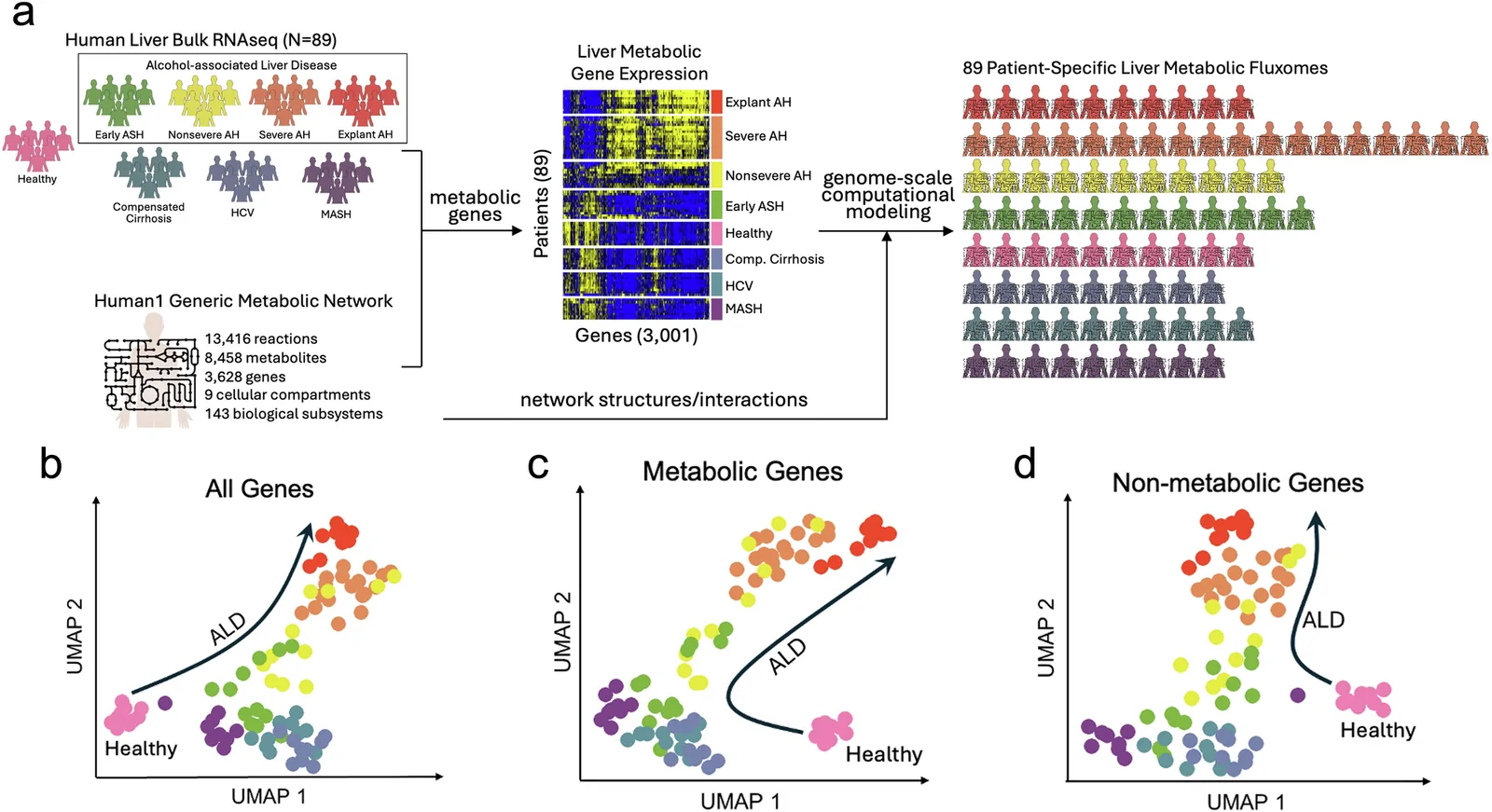
Workflow to predict patient-specific metabolic fluxomes and identify transcriptional variability among patients with liver disease. (**a**) Bulk RNAseq data of 89 liver samples from patients with varying alcohol-associated liver diseases, non-alcohol-associated liver diseases, and healthy controls are integrated with a generic human genome-scale metabolic model (Human1) to extract the metabolic genes. The patient-specific metabolic gene expression is then used as input along with the network structures and interactions from the Human1 model for genome-scale metabolic modeling and prediction of 89 patient-specific liver metabolic fluxomes. UMAP plots are shown for (**b**) all genes (N = 23,717), (**c**) metabolic genes alone (N = 3,001), and (**d**) non-metabolic genes alone (N = 20,716). The schematic human outlines in panel (a) were manually drawn in PowerPoint.

We utilized a systems-level genome-scale metabolic modeling approach to analyze the metabolic landscape of livers from patients with varying liver disease etiologies. The transcriptome from patients with ALD (early alcoholic steatohepatitis (early ASH), nonsevere alcoholic hepatitis (nonsevere AH), severe alcoholic hepatitis (severe AH), and explant alcoholic hepatitis (explant AH)), NALD (Compensated cirrhosis, hepatitis C virus (HCV), and metabolic dysfunction-associated steatohepatitis (MASH)) as well as healthy controls was subset to include only the metabolic genes present in the Human1 generic metabolic network (3,628 genes)^21^ (Fig. 1a). Of those genes in the network, 3,001 metabolic genes were detected and present in the RNAseq dataset across the patients. The metabolic gene expression, along with the network structures and interactions from the Human1 network (i.e., reaction-specific GPRs and cross-compartmental reactions), were then utilized for pruning the structure of each patient-specific genome-scale metabolic model for which metabolic fluxes could then be predicted for each the 89 patients. The patient-specific liver metabolic fluxomes were predicted using the Pheflux algorithm^22^, which predicts a single flux for each metabolic reaction for each patient, resulting in 13,416 flux predictions per patient (Fig. 1a).

### A gene-centric patient-specific analysis of liver disease states

We were interested in identifying patterns and variability within and across the patients in their respective disease state cohorts at the gene expression level by visualization of the RNAseq data in the form of a UMAP plot. First, the entire transcriptome (23,717 genes) was analyzed in a UMAP plot, which captured the distinct trajectory from the healthy state to the extreme cases of ALD (Fig. 1b). Specifically, the trajectory spanned from the healthy patient cohort, which exhibited the least amount of variability, to that of early ASH, nonsevere AH, severe AH, and terminating at explant AH. Patients with early ASH and nonsevere AH exhibited the most variability within their patient cohorts. Next, the transcriptome was subset to only metabolic genes, of which 3,001 of the total 3,628 genes from the generic Human1 model were nonzero across all patients. Similarly to the UMAP for the entire transcriptome, the UMAP plot containing metabolic genes alone also shows progression from the early to late stages of ALD (Fig. 1c). While the healthy patient samples still show little inter-cohort variability, they are more distinct from the ALD and NALD patient samples. Finally, the transcriptome was subset to the non-metabolic genes (20,716 genes), where the progression from early to late ALD is visible, however there is extensive variability among samples in each patient cohort (Fig. 1d). Therefore, we concluded that the metabolic gene expression was able to reproduce the patient-specific patterns and variability from the entire transcriptome, which is seen to a lesser extent when analyzing the non-metabolic gene expression.

### A systems-level analysis of predicted patient-specific metabolic fluxes

**Fig. 2:**
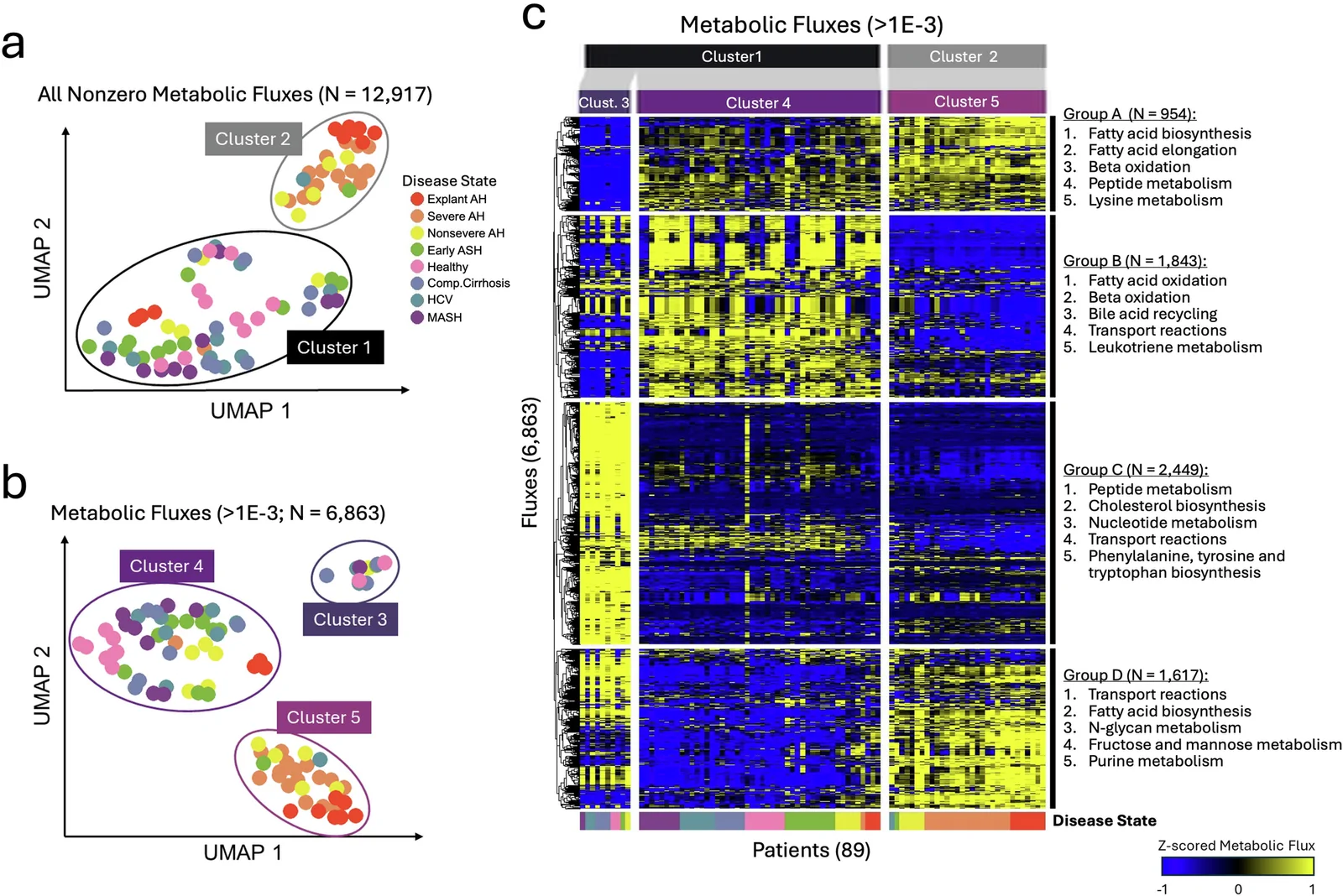
Systems-level analysis of patient-specific predicted metabolic fluxomes. (**a**) UMAP plot of all nonzero metabolic fluxes (N = 12,917) with K-means clustering (k = 2). (**b**) UMAP plot of all metabolic fluxes greater than the set threshold of 0.001 mmol/gDW/g (N = 6,863) with K-means clustering (k = 3). (**c**) Sankey plot showing the flow of patients from clusters in Fig. 2a to clusters in Fig. 2b (top). Hierarchical clustering of metabolic fluxes with cutoff > 0.001 mmol/gDW/g (N = 6,863) after z-scoring, resulting in 4 functional clusters (Group A-D). An overrepresentation analysis was performed for reactions within each of the functional clusters, and the top metabolic subsystems are shown.

The patient-specific predicted metabolic fluxomes were then analyzed at two levels of resolution, namely for all nonzero fluxes (N = 12,917; resolution level 1), and fluxes > 0.001 mmol/gDW/g cutoff (N = 6,863; resolution level 2). We analyzed the metabolic fluxes at each level of resolution and visualized them in the form of UMAP plots. Then, we performed k-means clustering to identify groups of patients with similar metabolic flux behavior and compared between the two levels of resolution. K-means clustering of all nonzero metabolic fluxes (resolution level 1) with k = 2, which was set based on the silhouette method for choosing the optimal k value (Supplementary Fig. 2a) resulted in two clusters, in which Cluster 1 contained the majority of samples (65.2%), including all healthy patients and most patients with NALD (96.4%) (Fig. 2a). Additionally, nearly all of the early ASH patient samples (91.7%), as well as roughly half of the nonsevere AH patient samples (54.5%), were in Cluster 1, while Cluster 2 contains most of the patients with extreme cases of ALD (94.4% of severe AH and 70% of explant AH patient samples). When analyzing metabolic fluxes greater than the 0.001 cutoff (resolution level 2), k-means clustering with k = 3, which was set based on the silhouette method for choosing the optimal k value (Supplementary Fig. 2b), resulted in three distinct groups (Fig. 2b). Cluster 3 contains a small mixed group of patient samples subset from Cluster 1 in the first level of resolution, including those from the following cohorts: MASH, HCV, Compensated cirrhosis, healthy, early ASH, and nonsevere AH. Cluster 4 is also a subset of Cluster 1 but contains the majority of samples (82.8% of samples from Cluster 1). Clusters 3 and 4 contain subsets of patients from Cluster 1 (resolution level 1), thereby highlighting the metabolic heterogeneity of the healthy and NALD patient samples within Cluster 1. Cluster 5, which contains most of the extreme cases of ALD, is identical to Cluster 2, which emphasizes the more consistently discrete metabolic flux activity within the ALD patient population despite the two levels of resolutions analyzed.

Further, a Sankey plot was generated to show the flow of information (i.e., the flow of patients) from the clusters at each level of resolution (Fig. 2c). As described above, Clusters 3 and 4 (resolution level 2) are subsets of Cluster 1 (resolution level 1), while Cluster 5 (resolution level 2) is identical to Cluster 2 (resolution level 1). To identify the functionality of the predicted metabolic fluxes from each of the patient clusters at the various resolutions, we performed hierarchical clustering of the metabolic fluxes that were greater than the 0.001 mmol/gDW/g cutoff (6863 fluxes) and then visualized them in the form of a heatmap (Fig. 2c). Hierarchical clustering of the fluxes resulted in 4 functional clusters of metabolic fluxes that define the unique functional behavior of each of the patient clusters previously identified from the UMAP plots. We then performed an overrepresentation analysis for each of the four functional clusters of metabolic fluxes to determine which subsystems are significantly overrepresented. Functional flux group A (N = 954) contains metabolic fluxes that are upregulated in Cluster 5 and, to a lesser extent, Cluster 4 and consists of reactions in the Fatty acid biosynthesis, Fatty acid elongation, Beta oxidation, Peptide metabolism, and Lysine metabolism metabolic pathways. Metabolic fluxes are upregulated in Cluster 4 in functional flux group B (N = 1843) and consist of reactions in the following metabolic pathways: Fatty acid oxidation, Beta oxidation, Bile acid recycling, Transport reactions, Leukotriene metabolism. Metabolic fluxes are upregulated in Cluster 3 of functional flux group C (N = 2449), which consists of reactions from subsystems including Peptide metabolism, Cholesterol biosynthesis, Nucleotide metabolism, Transport reactions, Phenylalanine, tyrosine and tryptophan biosynthesis. Functional flux group D (N = 1617) consists of fluxes upregulated in Clusters 3 and, to a lesser extent, Cluster 5, which are representative of reactions in the following metabolic subsystems: Transport reactions, Fatty acid biosynthesis, N-glycan metabolism, Fructose and mannose metabolism, Purine metabolism.

We were interested in determining how the metabolic flux annotations at the two levels of resolution map to the UMAP plot of metabolic genes and if the flux cluster annotations group together in the metabolic gene space. We found that the most discrete separation between groups occurred when mapping the nonzero flux cluster annotations (resolution level 1) onto the UMAP of metabolic genes (Supplementary Fig. 2c). This is because Cluster 2 (resolution level 1) contains the majority of patients with extreme cases of ALD, which exhibited distinct separation at the far end of the ALD trajectory in the UMAP space of metabolic genes alone (Fig. 1c). Since Cluster 5 (resolution level 2) is identical to Cluster 2 (resolution level 1), the samples from this group continue to show distinct separation from the rest of the clusters when overlaying the flux cluster annotations onto the UMAP of metabolic genes expression (Supplementary Fig. 2c, d). However, when overlaying the flux annotations from resolution level 2 onto the UMAP of metabolic genes, there are no distinct visual differences between Clusters 3 and 4 (Supplementary Fig. 2d). Therefore, the functional metabolic activity highlights additional heterogeneity between samples that is not identifiable at the metabolic gene level.

### Identifying metabolic flux activity associated with ALD progression

**Fig. 3:**
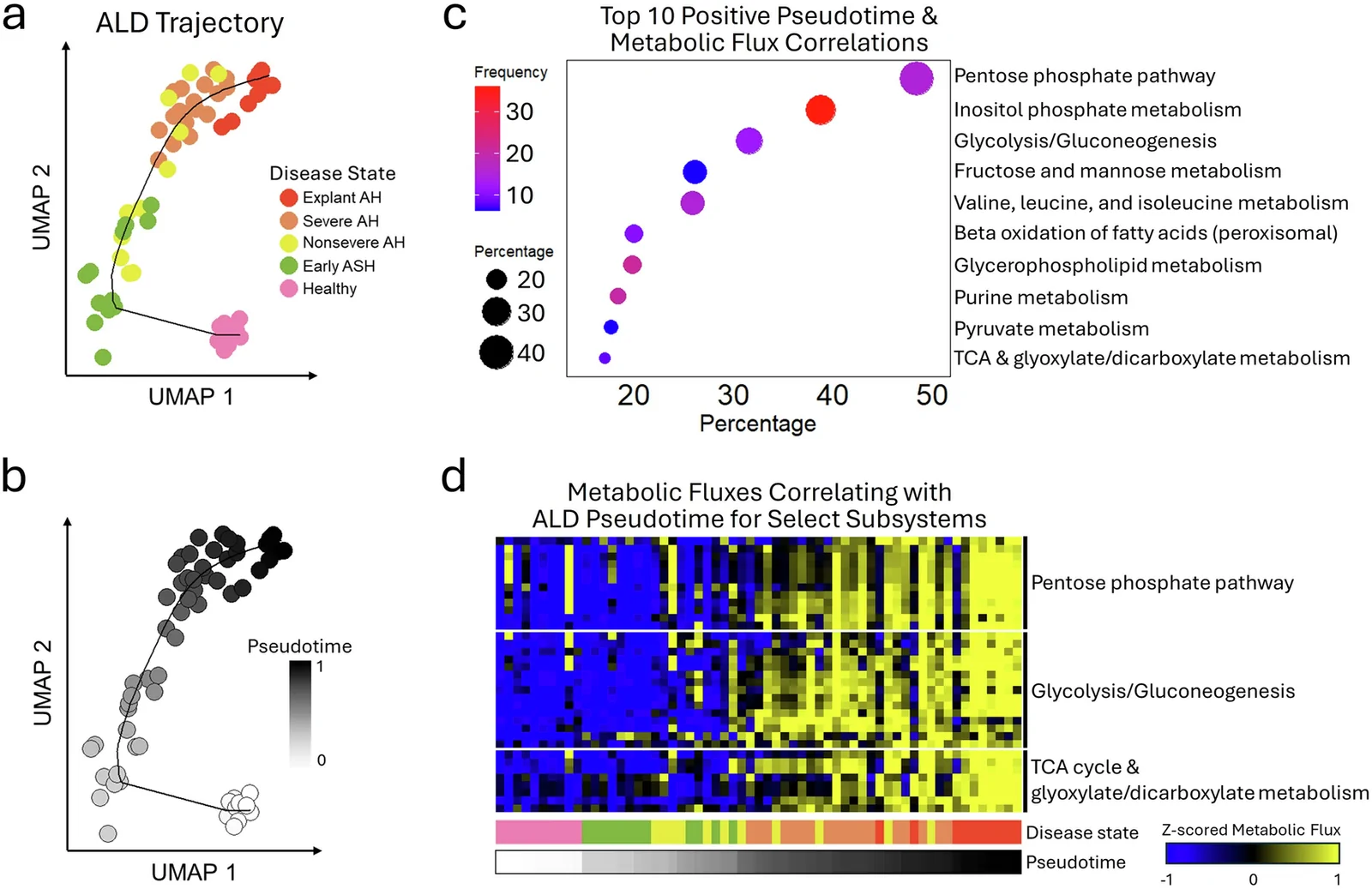
Metabolic flux correlations with ALD pseudotime. (**a**) ALD trajectory is shown on the UMAP of metabolic genes alone, subset to ALD and healthy control patient samples only. (**b**) Patient samples were pseudo-temporally ordered from healthy control (pseudotime = 0) to explant AH (pseudotime = 1) via Early ASH, Nonsevere AH, and Severe AH and was overlaid onto the UMAP of metabolic genes alone subset to ALD and healthy controls patient samples only. (**c**) The top 10 metabolic subsystems for which the associated metabolic fluxes for reactions within each of the subsystems positively correlate with the calculated pseudotime. (**d**) Metabolic fluxes that positively correlate with pseudotime from the pentose phosphate pathway, glycolysis/gluconeogenesis pathway, and TCA cycle & glyoxylate/dicarboxylate metabolic pathway are shown.

Because the regulatory and functional behavior of the patients with AH significantly differed from those with NALD, we were interested in determining which metabolic fluxes from the various metabolic subsystems showed correlations with the progressive nature of liver disease due to alcohol. To quantify the progression of individual patient samples along a trajectory from the healthy state to explant AH (via early ASH, nonsevere AH, and severe AH), we performed pseudo-temporal ordering of patient samples using the coordinates from the UMAP plot of only metabolic genes (Fig. 3a). We considered the first healthy patient sample to start at a pseudotime of 0 and the last explant AH patient sample ending at pseudotime 1 (Fig. 3b). We then identified reactions whose predicted metabolic fluxes positively correlated with pseudotime (spearman rho > 0.5 and *p*-value < 0.05). We tabulated these reactions by their representative metabolic subsystem and identified the top 10 subsystems with the highest number of correlated reactions per total number of reactions in the subsystem (Fig. 3c). The top subsystem was the Pentose phosphate pathway in which 15 (Frequency) out of the total 31 reaction fluxes (Percentage = 48.4%) in the subsystem correlated with the pseudo-temporal progression. Additional top subsystems include glycolysis/gluconeogenesis and tricarboxylic acid (TCA) cycle & glyoxylate/dicarboxylate metabolism. This was interesting as these three subsystems are highly interconnected and are involved in glucose utilization and energy metabolism. Plotting of the metabolic fluxes from the pentose phosphate pathway, glycolysis/gluconeogenesis, and TCA cycle & glyoxylate/dicarboxylate metabolism subsystems in the form of a heatmap show the significant upregulation of fluxes associated with pseudo-temporal ordering (Fig. 3d). Further, it is evident that there is a switch in metabolic flux activity from the healthy and early ALD patient samples to the extreme ALD patient samples in these metabolic subsystems related to energy metabolism. Therefore, we were interested in further analyzing the metabolic fluxes and associated gene expression data for the reactions in these pathways to identify how glucose utilization and energy metabolism may be altered with AH.

### Systems-level reprogramming of energetic metabolic pathways in AH

Previous work from our group has shown, using liver transcriptomics and metabolomics data, that there is specific metabolic reprogramming that occurs within the glycolysis pathway and TCA cycle that may promote shunting towards the pentose phosphate pathway in patients with AH^8^. As we have shown previously, the fluxes from reactions within the glycolysis, TCA cycle, and pentose phosphate pathways positively correlate with the pseudo-temporal progression of patient samples from the healthy patient cohort and along the ALD trajectory (Fig. 3). Therefore, this prompted us to further study the metabolic fluxes driving this glucose-mediated metabolic reprogramming.

**Fig. 4:**
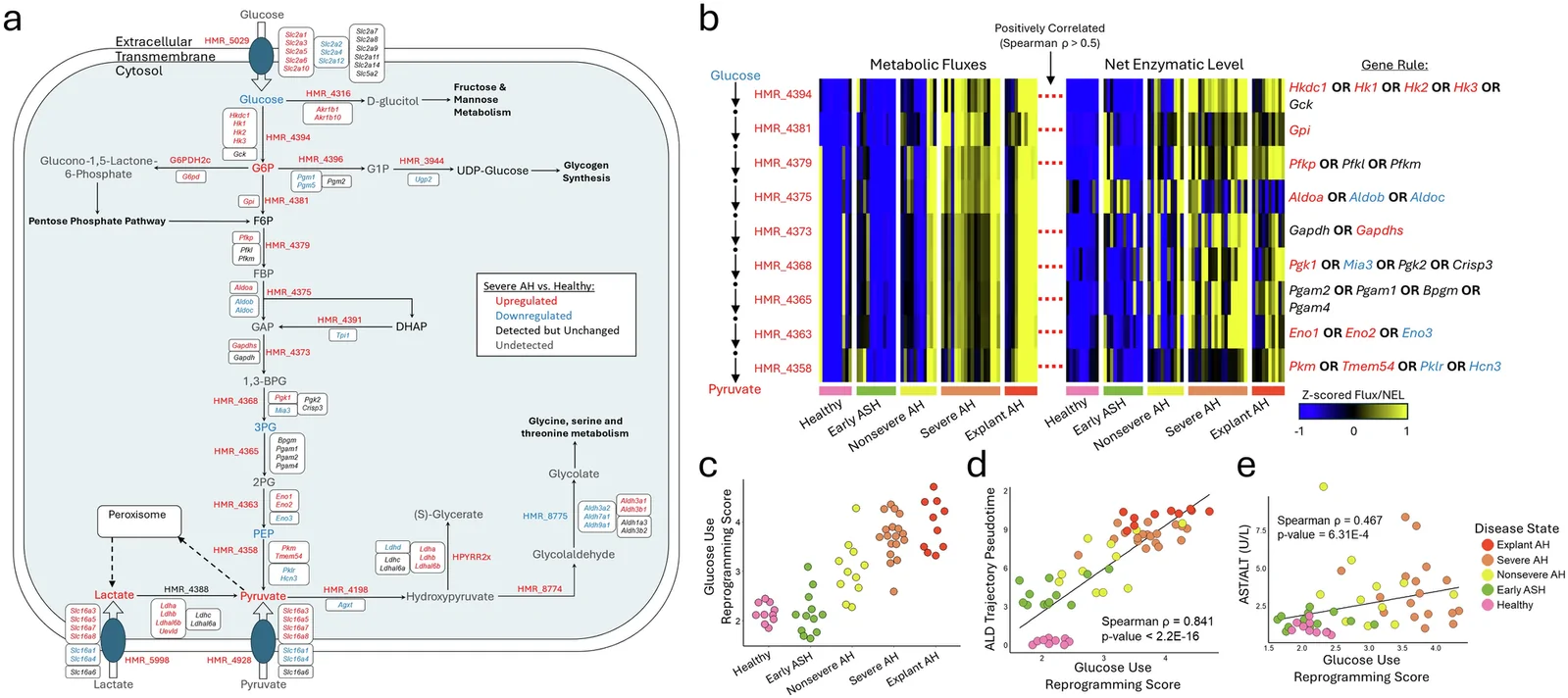
Glycolytic flux is upregulated in patients with AH and glucose use reprogramming scores correlate with disease progression and clinical parameters. (**a**) Glucose import to various pathways, including fructose and mannose metabolism, the pentose phosphate pathway, glycogen synthesis, and glycolysis are shown. Transcriptomics, predicted fluxomes, and publicly available metabolomics data^8^ are overlaid on the metabolic map and show upregulated (red), downregulated (blue), detected but unchanged (black), and undetected (gray) levels in the severe AH vs. healthy control patient cohorts. (**b**) Predicted metabolic fluxes and associated net enzymatic levels (NEL) are shown for the major reactions within the glycolysis pathway (i.e., glucose consumption to pyruvate production). Positive Spearman correlations (ρ > 0.5, p-value < 0.05) between the metabolic fluxes and NELs are shown in red. In addition, the gene rules encoding each reaction are displayed with the genes colored by severe AH vs. healthy changes (upregulated = red, downregulated = blue, detected but unchanged = black). (**c**) Glucose use reprogramming score increases according to disease progression. (**d**) Glucose use reprogramming scores significantly positively correlate with ALD trajectory pseudotime from Fig. 3A. (**e**) Glucose use reprogramming scores significantly correlate with patient AST/ ALT ratios.

We first wanted to establish a higher-level understanding of glucose utilization and shunting towards various pathways, including the pentose phosphate pathway, fructose and mannose metabolism, glycolysis, and glycogen synthesis. For all pathways, we found that the metabolic fluxes were upregulated in severe AH vs. healthy patients despite the gene expression changes (Fig. 4a). The majority of reactions within the glycolysis pathway have metabolic fluxes that are upregulated with severe AH relative to healthy controls and positively correlate (spearman rho > 0.5 and *p*-value < 0.05) with the calculated net enzymatic level (NEL) (Fig. 4b**;** see Methods). However, several fluxes are negatively correlated with their calculated NEL (spearman rho < -0.5 and *p*-value < 0.05), namely the glucose import flux (HMR_5029), the glucose-6-phosphate (G6P) → glucose-1-phosphate (G1P) flux (HMR_4396), and the pyruvate → hydroxypyruvate flux (HMR_4198) (Supplementary Fig. 3). Additionally, other fluxes show no correlation (0.5 > spearman rho > -0.5) with their calculated NEL (i.e., HMR_3944, HMR_4375, HMR_4391, HMR_4388, HMR_8774) (Supplementary Fig. 3). However, for the reactions whose metabolic flux either negatively or does not correlate with calculated NEL, we find that these fluxes are highly positively correlated with other fluxes in the glycolysis pathway. For instance, the metabolic fluxes for HMR_4396 are positively correlated with the metabolic fluxes for HMR_4394, HMR_4379, HMR_4375, HMR_4391, HMR_4373, HMR_4368, HMR_4365, HMR_4363, and HMR_4358 (all spearman rho > 0.5, *p*-value < 0.05).

Since there is an increase in glucose import and glycolytic flux in patients with AH, we sought to further understand how oxidative conditions and ATP generation via the TCA cycle and oxidative phosphorylation (OXPHOS) may be altered with disease. Glycolysis and the TCA cycle/OXPHOS are two major metabolic pathways that provide energy for cells. While energy production via glycolysis is faster and does not require oxygen, only 2 ATP molecules are produced^23^. Despite energy production via the TCA cycle and OXPHOS being slower and requiring oxygen, ~30 ATP molecules are produced^23^. Interestingly, we found that both O_2_ exchange with the environment (HMR_9048; severe AH vs. healthy FC = -1.12, pval = 4.57e-06) and O_2_ import from the extracellular space to the cytosol (HMR_4896; severe AH vs. healthy FC = -1.15, pval = 4.57e-06) are diminished with increasing severity of AH (Supplementary Fig. 4). Therefore, the livers of patients with severe AH are more hypoxic in comparison to healthy patient livers.

We sought to better understand how the hypoxic condition of the livers of patients with AH may contribute to energy production via the TCA cycle/OXPHOS. We find that the fluxes corresponding to reactions in the TCA cycle from alpha-ketoglutarate (AKG) towards the generation of fumarate (HMR_5295, HMR_4147, HMR_4152, HMR_4652) are upregulated in severe AH patient livers compared to healthy livers (Supplementary Fig. 5). Further, pyruvate consumption to acetyl-CoA (HMR_4137) and oxaloacetate (OAA) is also upregulated with AH. In contrast, fluxes corresponding to the reaction converting malate to OAA (HMR_4141) are downregulated in severe AH patients vs. healthy controls (Supplementary Fig. 5). All other metabolic fluxes encoding reactions in the TCA cycle are unchanged between patients with AH vs. healthy controls (Supplementary Fig. 5).

In accordance with the upregulated fluxes in the TCA cycle with AH, the first reaction of the OXPHOS pathway (HMR_6921: 5 H + [m] + NADH[m] + ubiquinone[m] => NAD + [m] + ubiquinol[m] + 4 H + [i]) is also upregulated with AH (Supplementary Fig. 6). However, the fluxes are significantly downregulated with AH in the intermediate steps of OXPHOS, thereby inhibiting energy production (Supplementary Fig. 6).

The pentose phosphate pathway also exhibits an upregulation of metabolic fluxes from G6P to F6P via the formation of intermediates including glucono-1,5-lactone-6-phosphate and 6-phosphogluconate, among others (Supplementary Fig. 7). F6P is then utilized in the glycolysis pathway towards the generation of pyruvate.

### Developing a glucose use reprogramming score using a machine learning model trained on glycolytic fluxes

Finally, we sought to generate a score of glucose use reprogramming that is associated with ALD progression and could accurately predict glycolytic flux phenotypes. We chose to develop a linear regression Support Vector Machine (SVM) model as it is relatively simple, deterministic, and can be applied to various testing datasets, allowing for the calculation of glucose use reprogramming scores with ease. Our training dataset consisted of the fluxes for reactions in the glucose import and glycolysis pathway (HMR_5029, HMR_4394, HMR_4381, HMR_4379, HMR_4375, HMR_4391, HMR_4373, HMR_4368, HMR_4365, HMR_4363, HMR_4358) for patients in the healthy, early ASH, nonsevere AH, and severe AH (including explant AH) disease cohorts (see Methods). We utilized the recursive feature elimination (RFE) algorithm with leave-one-out cross-validation (LOOCV) to identify the most pertinent predictors of disease state progression and used them in the generation of our final SVM model. The three reaction fluxes chosen to be included in the model were HMR_5029 (glucose import), HMR_4394 (glucose → G6P), and HMR_4381 (G6P → F6P). Interestingly, the metabolic flux features that met the criteria encode reactions at the beginning of the glycolysis pathway. Therefore, the metabolic fluxes for the reactions in the upstream portion of the glycolysis pathway appear to predict the downstream glycolytic flux activity and glucose use reprogramming phenotype of a patient.

The model was applied to all healthy patients and those with ALD (early ASH, nonsevere AH, severe AH, explant AH) to calculate a glucose use reprogramming score for each patient. The calculated scores show progression with ALD severity (Fig. 4c), a high positive correlation with ALD pseudotime (Fig. 4d; Spearman rho = 0.841, *p*-value < 2.2E-16), and a moderate correlation with AST/ALT levels (Fig. 4e; Spearman rho = 0.467, *p*-value = 6.31E-4). Additionally, the glucose use reprogramming scores mildly positively correlate with the following clinical parameters: 1) Model for End-stage Liver Disease (MELD) (spearman rho = 0.303, *p*-value = 0.0680), 2) Age, serum Bilirubin, International Normalized Ratio, and serum Creatinine (ABIC) (spearman rho = 0.297, *p*-value = 0.0743), and 3) Child–Pugh scores (spearman rho = 0.332, *p*-value = 0.0388) (Supplementary Fig. 8).

### *Hkdc1* expression drives glucose import toward glycolysis in the livers of patients with AH

**Fig. 5:**
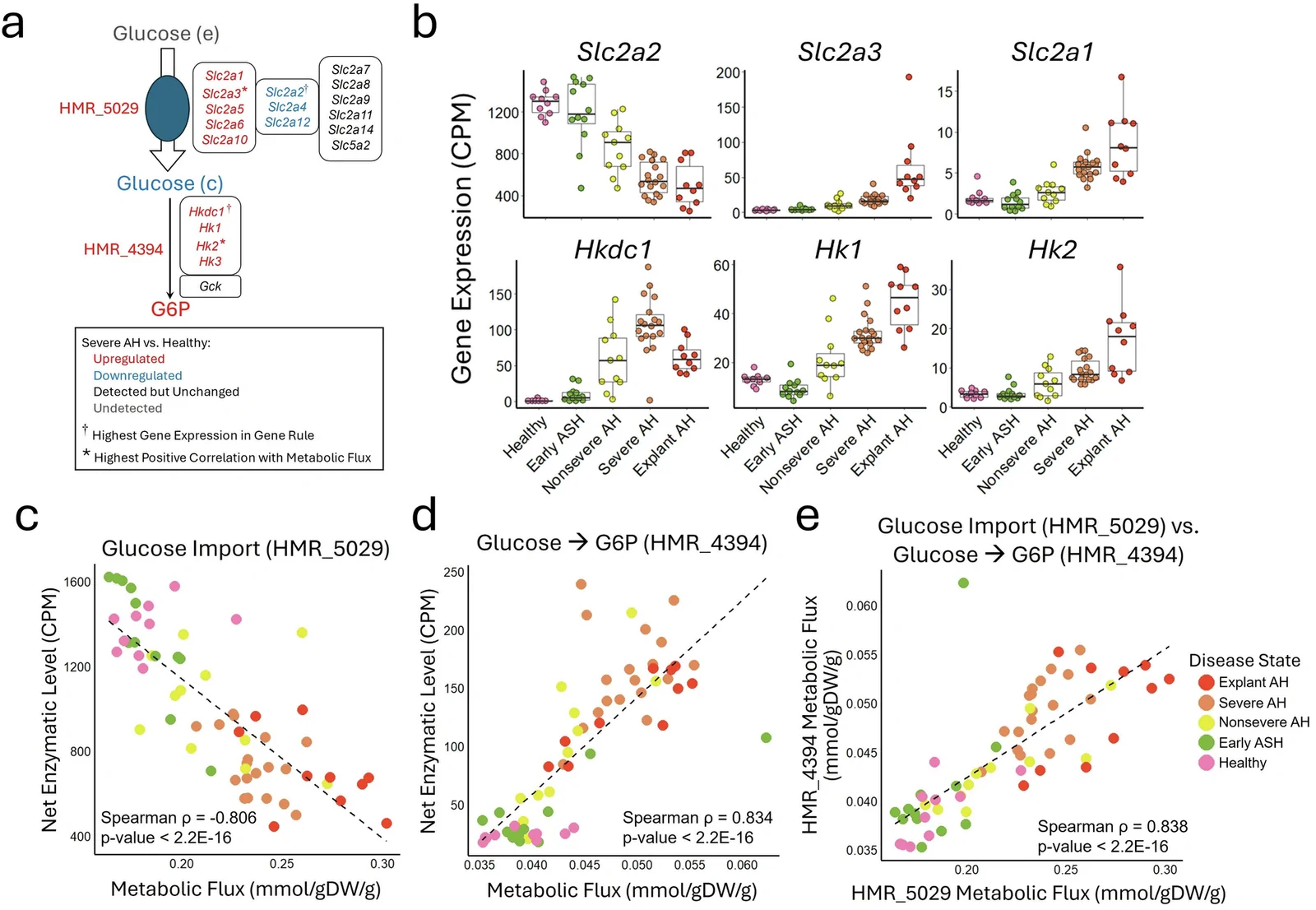
Metabolic behavior of upstream glucose import to glucose-6-phosphate production. (**a**) Transcriptomics, predicted fluxomes, and publicly available metabolomics data^8^ are overlaid on the metabolic map of glucose import (HMR_5029) and glucose → G6P (HMR_4394). Significant differences between Severe AH and healthy control patients are shown as follows: upregulated (red), downregulated (blue), detected but unchanged (yellow highlight), and undetected (no color). The gene with the highest absolute expression within the GPR of each reaction is denoted with a dagger (†), and the gene within the GPR with the highest positive correlation with the associated metabolic flux is denoted with an asterisk (*). (**b**) Gene expression patterns for the following genes: *Slc2a1, Slc2a3, Slc2a2, Hkdc1, Hk1, Hk2*. Scatter plots are shown for (**c**) glucose import (HMR_5029) NEL vs. metabolic flux, (**d**) glucose → G6P (HMR_4394) NEL vs. metabolic flux, and (**e**) glucose import (HMR_5029) metabolic flux vs. glucose → G6P (HMR_4394) metabolic flux.

Of specific interest was the glucose import reaction (HMR_5029), which is catalyzed by enzymes encoded by the GLUT transporter family and establishes the rate at which glucose enters the cytoplasm from the extracellular space (Fig. 5a). Under physiologically healthy conditions, *Slc2a2* is the main glucose transporter in the liver, as it has a low affinity but high capacity for glucose and plays an important role in glucose sensing and uptake for storage as glycogen^24–26^. Conversely, *Slc2a1* has a high affinity for glucose and is present at high concentrations in various other tissues for a constant supply of glucose for energy production^24–26^. *Slc2a3* is another glucose transporter that shares a high sequence similarity to *Slc2a1*, thereby also exhibiting a high affinity for glucose^24^. While the expression of *Slc2a1* and *Slc2a3* positively correlates with the HMR_5029 flux (Spearman rho = 0.802 and 0.865, respectively), *Slc2a2* expression negatively correlates with the flux (Spearman rho = -0.819) (Fig. 5a, b). Yet, *Slc2a2* has the highest overall expression of all genes in HMR_5029’s GPR, thus contributing most to the calculated NEL, which is also negatively correlated with flux (Fig. 5b, c). The literature suggests that with ALD, *Slc2a1* and *Slc2a3* expression increases and *Slc2a2* expression decreases, which is in line with our findings (Fig. 5b)^27,28^. One hypothesis for this dysregulation is that the altered glucose transporter expression accompanies the depletion of glycogen, reflecting a compensatory response to attenuated gluconeogenic activity to meet the increased intracellular demand for glucose^27^.

Following its import, glucose is utilized as a substrate to generate G6P (HMR_4394), which can then be utilized and shunted towards various other metabolic pathways, including glycolysis, glycogen synthesis, or the pentose phosphate pathway. The glucose → G6P reaction is catalyzed by enzymes encoding hexokinases. Our group and others have shown that upregulation of hexokinase *Hkdc1* with disease and cancer contributes to aberrant glucose import and metabolism^8,29–31^. The transcriptomics data indicates that hexokinases *Hkdc1*, *Hk1*, *Hk2*, and *Hk3* are all significantly upregulated in severe AH vs. healthy patient samples, with *Hkdc1* having the highest overall expression of all genes in the GPR and *Hk2* having the highest positive correlation with the HMR_4394 flux (Spearman rho = 0.807), followed closely by the positive correlation with *Hk1* (Spearman rho = 0.78) (Fig. 5a, b). Therefore, the calculated NEL for HMR_4394 is highly positively correlated with the predicted metabolic fluxes (Fig. 5d). Interestingly, the glucose import (HMR_5029) fluxes and the glucose → G6P (HMR_4394) fluxes are highly positively correlated, suggesting that the upregulation of fluxes driving glucose into the cytosol is necessary for continued increase in flux from glucose to G6P (Fig. 5e).

Our analysis of the hepatic glucose levels (i.e., cytosolic glucose) from the publicly available liver metabolomics data in Massey et al. 2021^8^ indicates a significant depletion in severe AH patients when compared to healthy controls (fold change = -2.16, *p*-value = 1e-3) when testing for significance using the Wilcoxon Ranked Sum test (Fig. 5a). Further, glucose-6-phosphate (G6P) levels are upregulated in severe AH patients vs. healthy control (fold change = 1.8, *p*-value = 0.04) (Fig. 5a). These findings are in line with our metabolic flux predictions, which suggest that glucose consumption is being driven towards the production of G6P.

### Glucose use reprogramming phenotype results in the reorganization of pyruvate flux balances in AH

**Fig. 6:**
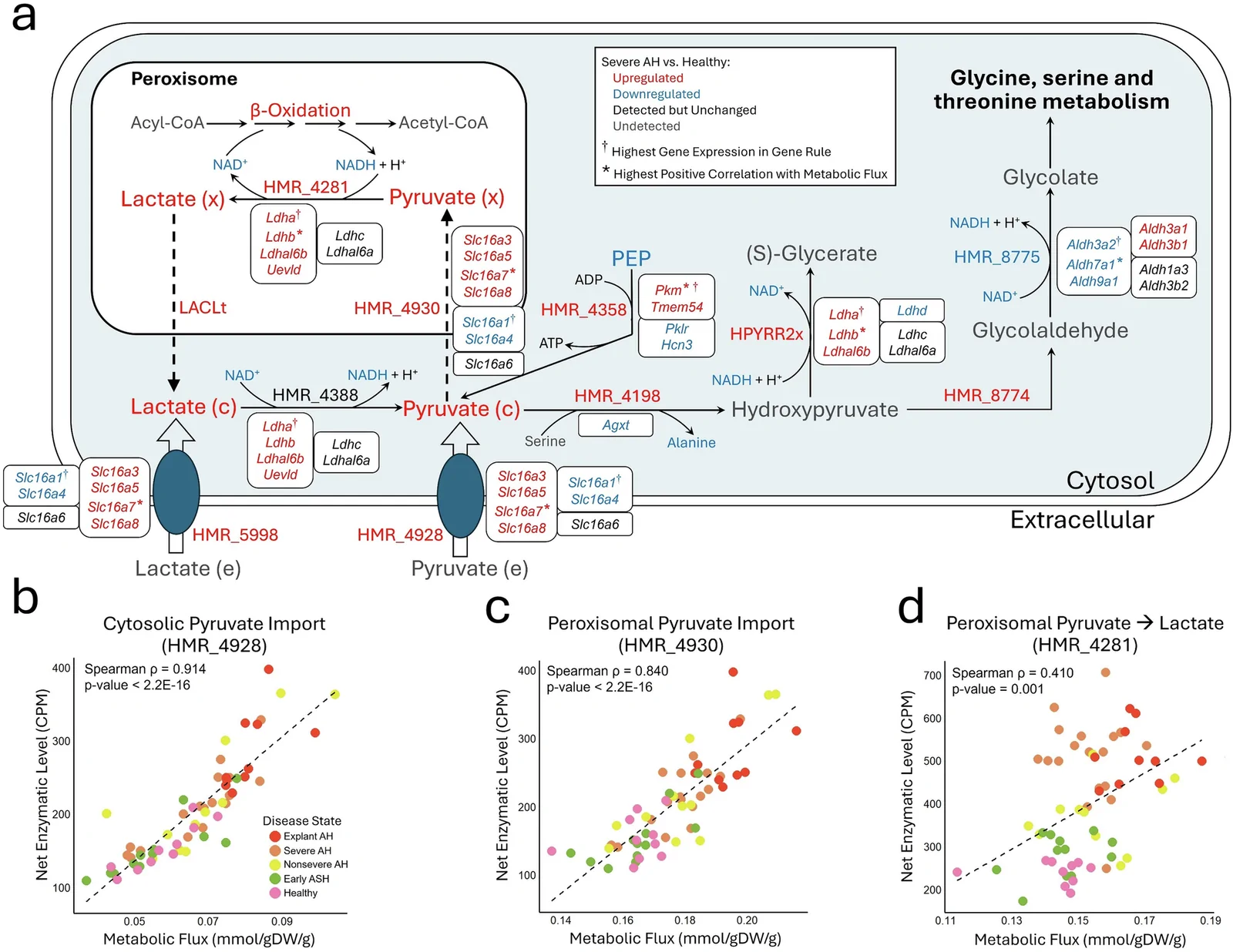
Pyruvate metabolic reprogramming with AH. (**a**) Transcriptomics, predicted fluxomes, and publicly available metabolomics data^8^ are overlaid on the metabolic map of reactions around the consumption and production of pyruvate. Significant differences between Severe AH and healthy control patients are shown as follows: upregulated (red), downregulated (blue), detected but unchanged (yellow highlight), and undetected (no color). The gene with the highest absolute expression within the GPR of each reaction is denoted with a dagger (†), and the gene within the GPR with the highest positive correlation with the associated metabolic flux is denoted with an asterisk (*). Scatter plots are shown for (**b**) cytosolic pyruvate import (HMR_4928) NEL vs. metabolic flux, (**c**) peroxisomal pyruvate import (HMR_4930) NEL vs. metabolic flux, and (**d**) peroxisomal pyruvate→ lactate (HMR_4281) NEL vs. metabolic flux.

The final reaction of glycolysis is the generation of pyruvate from phosphoenolpyruvate (PEP) (HMR_4358). The predicted metabolic fluxes converting PEP to pyruvate (HMR_4358) are upregulated in severe AH vs. healthy control patients and are nearly exclusively driven by the expression of *Pkm*, which is also upregulated with severe AH, is present at the highest overall level in the HMR_4358 GPR and is positively correlated with the HMR_4358 flux (Fig. 6a). Because of the significantly increased flux from PEP to pyruvate (HMR_4358), as well as the upregulated pyruvate import flux from the extracellular space to the cytosol (HMR_4928), the pyruvate metabolite levels in the liver tissue are subsequently upregulated. Intracellular pyruvate can then be utilized in various ways, i.e., shunting into the peroxisome or as a substrate for hydroxypyruvate production (Fig. 6a). If pyruvate is utilized as a substrate towards the generation of hydroxypyruvate, the hydroxypyruvate may then be converted to (S)-glycerate (L-glycerate) by reduction with lactate dehydrogenase and NADH, which has also been shown experimentally to occur in rat livers^32,33^. The metabolic fluxes are upregulated with AH, therefore driving towards the production of (S)-glycerate, which cannot be further metabolized, and NAD + , which can then be readily utilized for other reactions. Hydroxypyruvate can also be converted to glycolaldehyde, which is then utilized as a substrate for generating glycolate, a metabolite involved in glycine, serine and threonine metabolism (Fig. 6a). The fluxes for the hydroxypyruvate → glycolaldehyde reaction (HMR_8774) are upregulated in severe AH but concurrently downregulated for glycolaldehyde → glycolate production (HMR_8775), as the aldehyde dehydrogenases with the highest expression in the GPR (*Aldh3a2*) and highest positive correlation with the flux (*Aldh7a1*) are both downregulated with AH.

Metabolic fluxes for pyruvate and lactate import from the extracellular space to the cytosol (HMR_4928 and HMR_5998, respectively) as well as pyruvate import from the cytosol to the peroxisome (HMR_4930) are upregulated with severe AH due to the significantly positive correlations between the fluxes and calculated NEL’s (Fig. 6b-d). This is primarily due to the *Slc16a7* expression in the GPRs, which is most positively correlated with the metabolic fluxes (Fig. 6a). The increased pyruvate uptake flux into the peroxisome is then utilized for lactate production (HMR_4281) via various lactate dehydrogenases (most notably *Ldha* and *Ldhb*). Consumption of pyruvate, NADH, and H+ to produce lactate and NAD+ (HMR_4281) is coupled with various reactions in the B-oxidation pathway (Fig. 6a). Some reactions in the B-oxidation pathway that are coupled to HMR_4281 include HMR_3059 (3-hydroxyhexacosanoyl-CoA[x] + NAD + [x] => 3-oxohexacosanoyl-CoA[x] + H + [x] + NADH[x]), HMR_3096 ((S)-hydroxyoctanoyl-CoA[x] + NAD + [x] => 3-oxooctanoyl-CoA[x] + H + [x] + NADH[x]), HMR_3362 ((S)-3-hydroxy-11-cis-octadecenoyl-CoA[x] + NAD + [x] => 3-oxo-11-cis-octadecenoyl-CoA[x] + H + [x] + NADH[x]), etc., which utilize NAD+ towards the generation of end product acetyl-CoA, and are upregulated with severe AH. Lactate can also be shuttled out of the peroxisome back to the cytosol, where it can then be converted back to pyruvate via lactate dehydrogenases (Fig. 6a). Therefore, in livers of patients with AH, there is metabolic cycling of pyruvate and lactate: (1) pyruvate uptake to the peroxisome (HMR_4930), (2) conversion to lactate (HMR_4281), (3) export of lactate to the cytosol (LACLt), and (4) conversion back to pyruvate (HMR_4388) (Fig. 6a). Further, because the metabolic fluxes are unchanged for pyruvate generation from lactate in the cytosol (HMR_4388) and there is upregulation of pyruvate and lactate import from the extracellular space to the cytosol (HMR_4928 and HMR_5998, respectively) with AH, there is an excessive accumulation of hepatic lactate and pyruvate metabolite levels (Fig. 6a). We, therefore, identify pyruvate as a key player contributing to the reorganization of flux balances in energy metabolism pathways associated with AH.

### Identifying distinct severe AH patient groups following in silico gene knockdown intervention

**Fig. 7:**
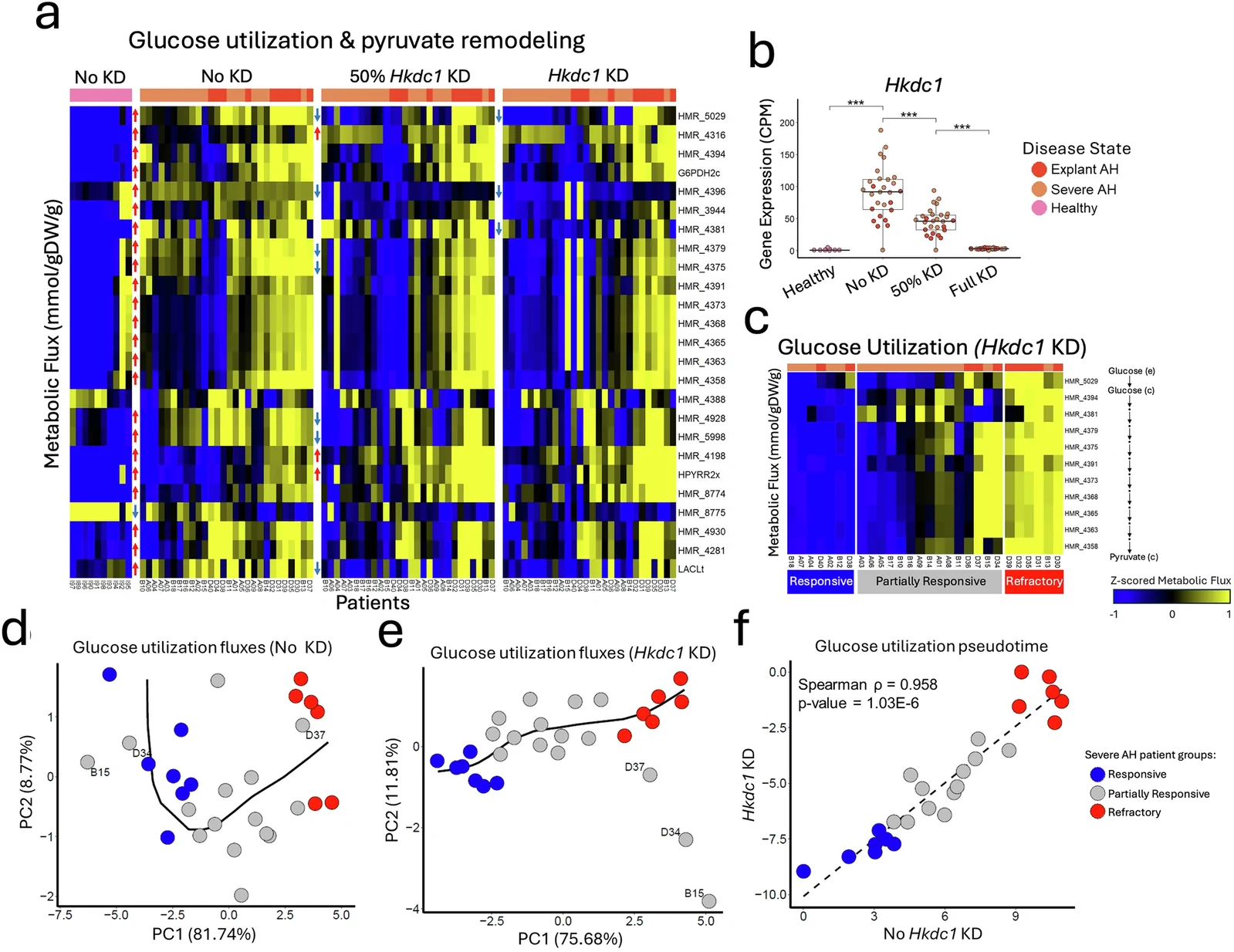
*Hkdc1* knockdown results in distinct patient-specific glycolytic phenotype that correlated with pseudotime. (**a**) Glucose import, glycolysis, and pyruvate metabolism reaction fluxes are z-scored and plotted in a heatmap for healthy patients and those in the severe AH and explant AH patient cohorts for no gene knockdown, 50% *Hkdc1* knockdown, and full *Hkdc1* knockdown within the range of healthy patients within the range of healthy patients. Significant differences between groups are shown with red up arrows (upregulated) and blue down arrows (downregulated). (**b**) *Hkdc1* expression in healthy patients and those within the severe AH and explant AH cohorts with no gene knockdown, 50% knockdown, and full knockdown within the range of healthy patients. (**c**) Hierarchical clustering of metabolic fluxes in the glycolysis pathway from glucose import to pyruvate production following Hkdc1 knockdown reveal three distinct subsets of patient responses: Responsive (blue), Partially Responsive (gray), and Refractory (red). The glucose utilization metabolic fluxes (**d**) prior to and (**e**) following *Hkdc1* knockdown were utilized for PCA, and the calculated pseudotemporal trajectory was overlaid, highlighting the progression from Responsive to Partially Responsive to Refractory. (**f**) A significant positive correlation exists between the pseudotime values for glucose utilization metabolic fluxes prior to and following *Hkdc1* KD. KD = knockdown. Significance testing: * p-value < 0.05, ** p-value < 0.01, *** p-value < 0.001.

We next sought to identify how the dysregulation of metabolic fluxes, specifically with respect to those around glucose import, glycolysis, and pyruvate remodeling, may be rebalanced such that they are adjusted back towards healthy ranges. Since we have shown that *Hkdc1* plays an imperative role in driving glucose use reprogramming in livers of patients with AH, we performed in silico knockdown (KD) experiments in which we adjusted the previously upregulated *Hkdc1* levels in the severe AH and explant AH patient cohorts to 50% of their original levels (50% KD) and also within the healthy patient range (full KD) and then re-analyzed the resulting glycolysis and pyruvate remodeling fluxes (Fig. 7a). The gene expression values following 50% and full *Hkdc1* knockdown are significantly reduced from the original severe AH and explant AH gene expression prior to knockdown (Fig. 7b). We found that most of the fluxes that were altered with *Hkdc1* knockdown have already changed in the direction of the healthy patient flux cohort with 50% *Hkdc1* knockdown, including the glucose import flux (HMR_5029) and other reactions in the main portion of the glycolysis pathway including HMR_4396: G6P[c] → G1P[c], HMR_4379: ATP[c] + F6P[c] → ADP[c] + FBP[c] + H + [c], and HMR_4375: FBP → DHAP[c] + G3P[c] (Fig. 7a). However, with the full *Hkdc1* KD, the glucose import fluxes are further reduced as is HMR_4396, while HMR_4381: G6P[c] → F6P[c] is unchanged with 50% *Hkdc1* KD but is significantly reduced with full *Hkdc1* KD (Fig. 7a).

Interestingly, we found three distinct subsets of severe AH patients following the full knockdown of *Hkdc1* when analyzing the fluxes from the reactions within the glycolysis pathway from glucose import to the production of pyruvate (Fig. 7c). Specifically, all of the main glycolysis fluxes (HMR_5029, HMR_4394, HMR_4379, HMR_4375, HMR_4373, HMR_4368, HMR_4365, HMR_4363, and HMR_4358) were downregulated in the Responsive patient cohort vs. the Refractory patient cohort in response to *Hkdc1* knockdown. We next performed PCA on the patients prior to (Fig. 7d) and following (Fig. 7e) *Hkdc1* knockdown using only these main glycolysis fluxes and plotted the first two components, annotating the patients by their respective subgrouping from Fig. 7c. Additionally, we performed pseudo-temporal trajectory analysis on the first two principal components and found that in both cases there exists a progression in glucose use reprogramming from patients beginning in the Responsive cohort and terminating at the Refractory cohort, with intermediate patients in the Partially Responsive cohort. We then generated a scatterplot of the pseudotime values before vs. after *Hkdc1* knockdown and found that there was a significantly positive correlation (spearman rho = 0.958, *p*-value = 1.03E-6) (Fig. 7f), indicating that the patients within the severe AH and explant AH exist along a continuum with respect to glucose use reprogramming.

**Fig. 8:**
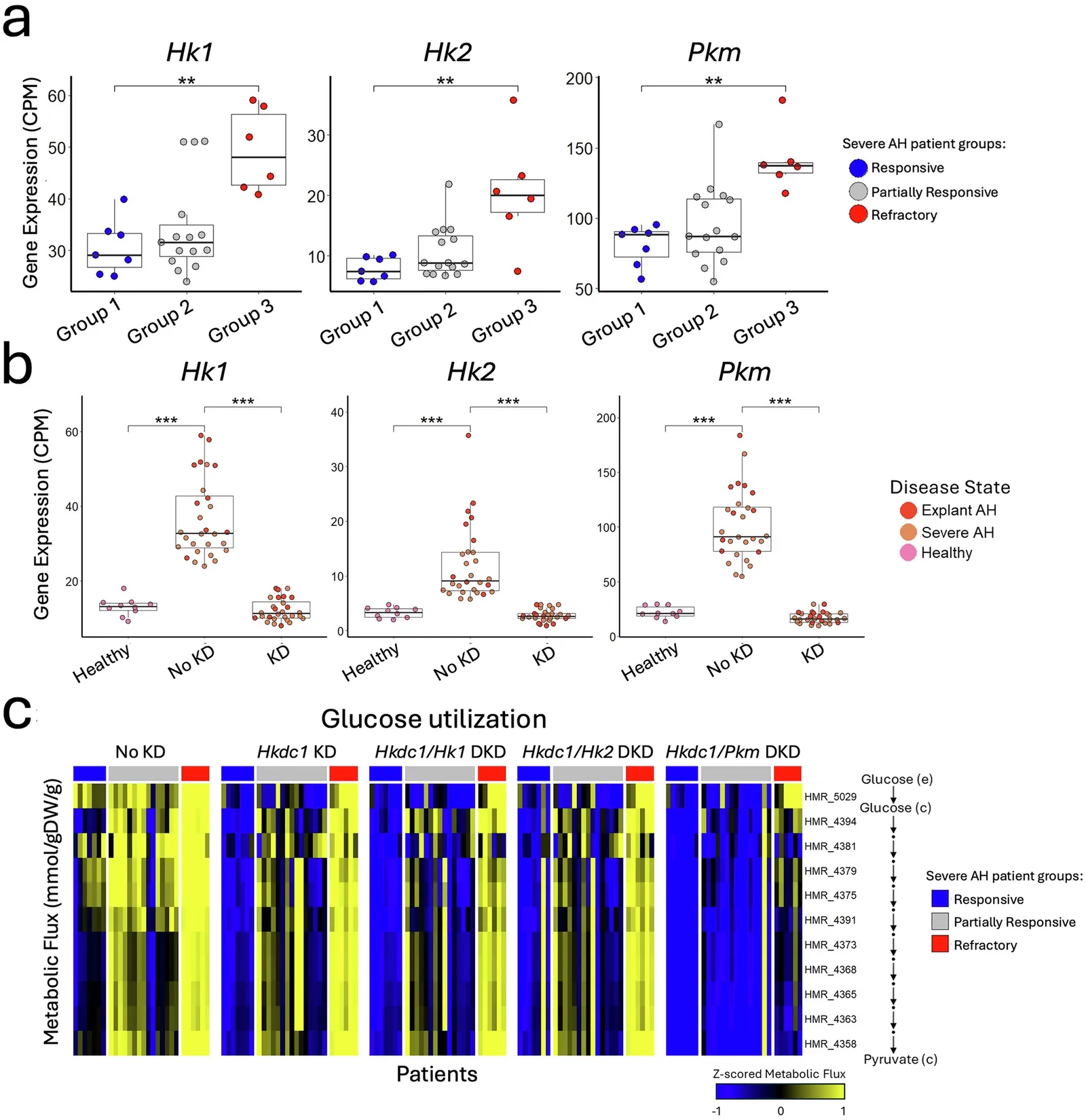
*Hkdc1*/*Pkm* double knockdown readjusts the glycolytic fluxes towards the healthy phenotype in all patients. (**a**) There is a significant upregulation of *Hk1*, *Hk2*, and *Pkm* expression from patients in the Responsive cohort to those in the Refractory cohort. (**b**) *Hk1*, *Hk2*, and *Pkm* expression are upregulated in patients within the severe AH and explant AH cohorts relative to healthy controls. This expression is abrogated upon knockdown of the genes within the healthy control range. (**c**) Following double knockdown of *Hkdc1*/*Hk1* and *Hkdc1*/*Hk2*, the patients within the Refractory cohort and a subset of patients in the Partially Responsive cohort retain their upregulated glycolytic fluxes, while double knockdown of *Hkdc1* and *Pkm* results in a robust downregulation of glycolytic fluxes in all patients. KD = knockdown, DKD = double knockdown. Significance testing: NS = not significant, * p-value < 0.05, ** p-value < 0.01, *** p-value < 0.001.

While there did exist significant downregulation of glucose use fluxes within each patient group following *Hkdc1* knockdown, the flux activity of patients within the Refractory patient cohort was still in range with other severe AH patient fluxes prior to knockdown and were upregulated relative to the glycolytic fluxes in the nonsevere AH patient cohort. Therefore, we sought to identify other possible genes that may be driving this flux behavior in patient group 3. We performed unbiased differential expression analysis between patients in the Responsive vs. Refractory cohorts and identified three genes within the glycolysis pathway, namely *Hk1*, *Hk2*, and *Pkm*, that were significantly upregulated in patients within the Refractory cohort relative to those in the Responsive cohort (Fig. 8a). As a control, we performed a series of additional in silico knockdown experiments of various other genes within the glycolysis pathway, including *Slc2a1*, *Slc2a3*, *Eno1*, *Gapdh*, *Ldha*, *Ldhb*, *Pfkp*, and *Pgk1* and found there to be no significant downregulation of glycolytic fluxes in any of the patient groups (Supplementary Fig. 9). We next performed combinatorial in silico knockdown experiments for *Hkdc1/Hk2*, *Hkdc1/Hk2*, and *Hkdc1/Pkm* to determine if patients in the Refractory cohort had a more robust response to these knockdown experiments, resulting in their glycolytic fluxes shifting more significantly towards a healthy phenotype. The gene expression of *Hk1*, *Hk2*, and *Pkm* was separately knocked down within the healthy range (Fig. 8b), the liver metabolic fluxomes were predicted using Pheflux, and the glucose utilization fluxes were analyzed. We found that only the combinatorial knockdown of *Hkdc1* and *Pkm* resulted in a significant reduction in glucose use metabolic flux activity in patients within the Refractory cohort (Fig. 8c). While the metabolic fluxes for glucose import remained high in patients in the Refractory cohort, the remaining metabolic fluxes in the glycolysis pathway were rebalanced towards the healthy phenotype.

### Decision tree using glycolytic gene expression and glucose use reprogramming score using glycolytic flux accurately predict patient response to intervention via in silico gene knockdown

**Fig. 9:**
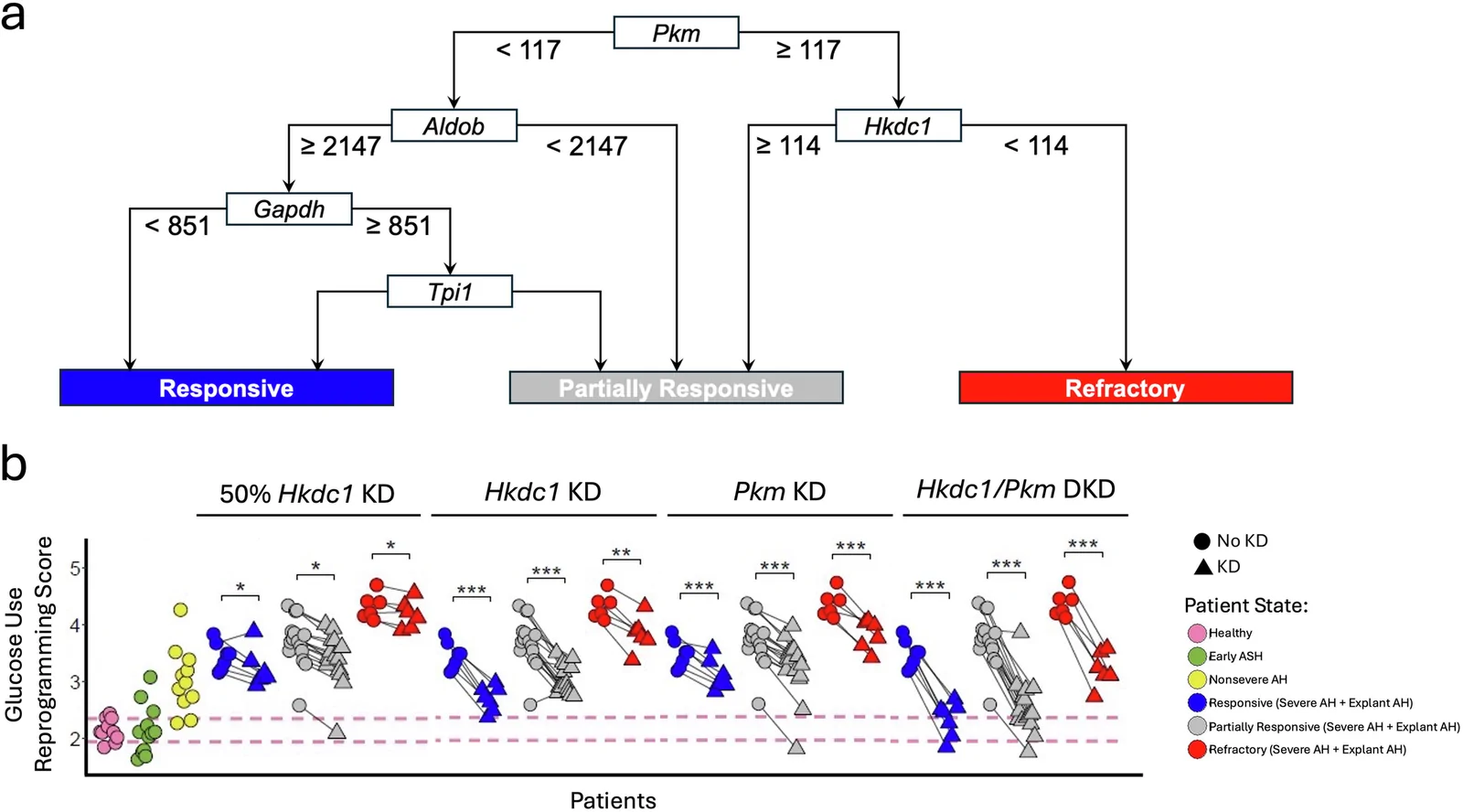
Decision tree trained on glycolytic gene expression and glucose use reprogramming score trained on glycolytic metabolic fluxes both accurately predict patient responses. (**a**) Decision tree trained on main glycolytic genes accurately captures the patient-specific responses (Responsive vs. Partially Responsive vs. Refractory) following *Hkdc1* knockdown. (**b**) Glucose use reprogramming scores are significantly upregulated in all patient response groups relative to healthy controls following 50% *Hkdc1* knockdown, full *Hkdc1*, and *Pkm* knockdown; however, only following *Hkdc1*/*Pkm* double knockdown, do the patients fall within the range of the healthy control patients. KD = knockdown, DKD = double knockdown. Significance testing: NS = not significant, * p-value < 0.05, ** p-value < 0.01, *** p-value < 0.001.

A decision tree was generated to test the predictive power of the glycolytic gene expression in classifying the three patient groups in response to *Hkdc1* knockdown. Only the main glycolysis genes with the most dominant expression for each reaction from glucose import (HMR_5029) to pyruvate production (HMR_4358) were provided to the decision tree model. These genes included *Slc2a2, Hkdc1, Hk1, Gpi, Pfkl, Pfkp, Aldob, Tpi1, Gapdh, Pgk1, Pgam1, Eno1, Pkm*, and *Pklr*. The final glycolysis genes included in the decision tree that were utilized for the classification of patient-specific glycolytic phenotypes were *Pkm, Hkdc1, Aldob, Gapdh*, and *Tpi* (Fig. 9a). We achieved 96.4% accuracy, with only one sample (patient B12) in the Responsive patient cohort misclassified as part of the Partially Responsive patient cohort as patient B12 has *Aldob* expression less than 2417 CPM (patient B12 *Aldob* expression = 1517.09 CPM).

At the level of gene expression, the decision tree was able to accurately classify the patient-specific glycolytic responses to *Hkdc1* knockdown. We next utilized the previously developed linear SVM regression model to test the patient’s glucose use reprogramming scores following in silico gene knockdown experimentation, which are calculated using the metabolic fluxes. We found that the calculated glucose use reprogramming scores are significantly reduced for all three patient subgroups following 50% *Hkdc1* knockdown, full *Hkdc1* knockdown, *Pkm* knockdown, and the combinatorial knockdown of *Hkdc1* and *Pkm* (Fig. 9b). However, only upon double knockdown of *Hkdc1* and *Pkm* do the glucose use reprogramming scores adjust in range with the scores of healthy control individuals, thus significantly rebalancing the glucose utilization fluxes (Fig. 9d). We found no significant differences in glucose use reprogramming scores for patients in the Responsive, Partially Responsive or Refractory cohorts following individual knockdown of *Hk1, Hk2, Slc2a1, Slc2a3, Eno1, Gapdh, Ldha, Ldhb, Pfkp*, or *Pgk1* (Supplementary Fig. 10).

**Fig. 10:**
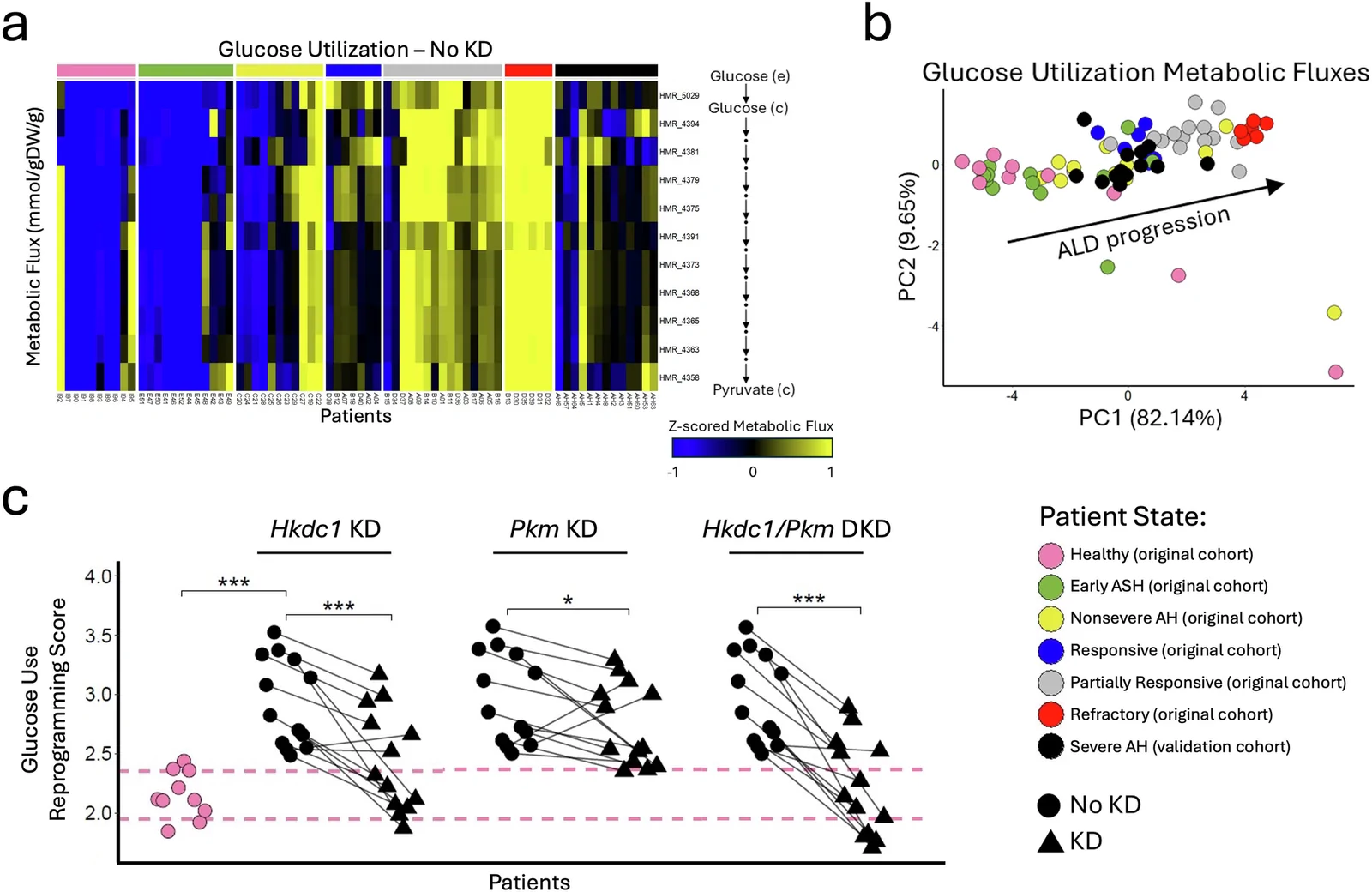
Validation of glucose use reprogramming scoring metric in the validation dataset (GSE143318). (**a**) The glucose utilization fluxes across patient groups, including the severe AH validation cohort, are shown in the heatmap. (**b**) The glucose utilization fluxes were used for PCA, and we identified the severe AH patient samples within the validation cohort clustering more closely with the patients in the Responsive and Partially Responsive patient groups from the original cohort. (**c**) *Hkdc1* knockdown alone as well as combinatorial knockdown of *Hkdc1* and *Pkm* resulted in significantly lower glucose use reprogramming scores that fell within the range of the healthy control patients. *Pkm* knockdown alone did not bring the glucose use reprogramming scores in range with the healthy individuals. KD = knockdown, DKD = double knockdown. Significance testing: NS = not significant, * p-value < 0.05, ** p-value < 0.01, *** p-value < 0.001.

In order to validate our method of glucose use reprogramming scoring, we utilized a publicly available, independent bulk RNAseq dataset consisting of 13 liver samples from patients with severe AH^34^ (GSE143318). Again, we utilized Pheflux to predict the metabolic fluxomes of the 13 severe AH patients in our validation cohort. To directly compare the metabolic fluxes from the original cohort with those in the validation cohort, we merged all fluxes into one matrix and corrected the entire fluxomes across batches (original vs. validation cohort). When visualizing the glucose use metabolic fluxes from glucose import to pyruvate production for patients in both the original cohort and the validation cohort following in the form of a heatmap, we identified that the severe AH patients in the validation cohort more closely mimic the metabolic flux behavior of patients within the Responsive group of the original cohort (Fig. 10a). To confirm this assumption, we performed PCA and found that the severe AH patients in the validation cohort clustered more closely with the patients in Responsive and Partially Responsive groups of the original cohort (Fig. 10b).

We then calculated a glucose use reprogramming score for each of the severe AH patients within the validation cohort and found there to be a significant upregulation from the healthy controls to the severe AH patients in the validation cohort (Fig. 10c). However, like in the case of severe AH patients within the Responsive group of the original cohort, the severe AH patients in the validation cohort robustly responded to *Hkdc1* knockdown alone, with glucose use reprogramming scores significantly reduced to levels within the healthy control range (Fig. 10c). Further, the glucose use reprogramming scores for the severe AH patients in the validation cohort following combinatorial knockdown of *Hkdc1* and *Pkm* but not *Pkm* knockdown alone also significantly reduced glucose use programming scores to levels within the healthy control range (Fig. 10c). Therefore, in accordance with the metabolic flux behavior of severe AH patients from the Responsive group of the original cohort, the glucose use reprogramming can be rebalanced in all patients with severe AH in the validation cohort when *Hkdc1* expression is knocked down, without the requirement of additional *Pkm* knockdown. Altogether, the glucose use reprogramming score highlights a unique opportunity to utilize metabolic fluxes to predict responses to AH interventions in a patient-specific manner.

## Discussion

While previous literature, in addition to our findings from Manchel et al. 2022^20^, has shown that there is metabolic reprogramming associated with ALD^8,9,11,20^, this study provides us with an extensive and comprehensive understanding of glucose use reprogramming in patients with AH via integration of multiple methodologies including transcriptomics, metabolomics, genome-scale metabolic modeling, flux balance analysis, and machine learning modeling. In this study, we highlight *Hkdc1* and *Pkm* as two significant drivers of glucose use metabolic reprogramming in patients with AH. Further, we propose that the engineering of these genes may open up a new therapeutic avenue for patients with AH.

Our systems-level approach to studying global patient-specific gene expression changes resulted in our finding of distinct trajectories at both the metabolic and non-metabolic levels from the healthy state to severe ALD (severe AH and explant AH) states. However, we found that additional variability exists between samples within a disease state when analyzing the non-metabolic genes compared to the metabolic genes. Interestingly, this patient trajectory of metabolic genes closely mimicked that of the entire transcriptome, underlying the important contribution of metabolism towards the overall transcriptomic profile of the liver.

The metabolic gene expression was integrated with the Human1^21^ metabolic network, and genome-scale metabolic modeling methodologies and flux balance analysis were performed using Pheflux^22^, resulting in the prediction of 89 patient-specific liver metabolic fluxomes. We chose to utilize the newly developed Pheflux algorithm as it does not require an objective function and does not depend on a correlative relationship between the fluxome and transcriptome. Instead, the Pheflux algorithm minimizes a statistical distance (the forward Kullback-Leiber divergence) between the fluxome and transcriptome, resulting in a predicted fluxome that minimizes the expected excess surprise with regard to the transcriptome^22^.

We then took a systems-level approach to analyze the predicted metabolic fluxes and found there to be an explicit distinction between the severe ALD samples (severe AH and explant AH) and all other patient samples including those from the healthy state, NALD states (comp. cirrhosis, HCV, and MASH), and less severe ALD states (early ASH, nonsevere AH). Patients with severe ALD exhibited increased predicted metabolic fluxes in fatty acid biosynthesis and elongation, peptide metabolism, N-glycan metabolism, and fructose and mannose metabolism pathways, alongside decreased fluxes in bile acid recycling, cholesterol biosynthesis, and nucleotide metabolism. This increase in fatty acid metabolism with ALD has been observed in both human and mouse studies, the mechanism of which is due to the combinatorial effect of increased glycerolipid synthesis, decreased fatty acid oxidation, and alteration of fatty acid export processes^35–38^. Chronic alcohol consumption has also been shown to disrupt cholesterol metabolism leading to ALD progression^39–41^. Further, we found that the ALD patient pseudotime trajectory based on the metabolic gene expression significantly positively correlated with predicted metabolic fluxes in the pentose phosphate pathway, glycolysis/gluconeogenesis pathway, and the TCA cycle. Consistent with our findings, Huang et al. 2021 identified a metabolomic fingerprint indicating that disruptions in metabolites associated with cellular energy supply contribute to the development and progression of alcohol-associated cirrhosis^42^. However, the global patterns that correlate with ALD progression do not readily separate into causal vs. consequential as the metabolic changes observed are systemic, affecting other organs and biological processes in the body. Additionally, there is feedback to the liver resulting in further disease progression. Therefore, trying to parse out these causal vs. consequential effects in the data is quite difficult without additional perturbation information or a substantial increase in the patient cohort size, thereby including naturally occurring perturbations. Despite this caveat, some metabolic shifts that occur in the less severe disease states (i.e., early ASH) are more likely to be causative based on known biology of ALD, such as that of glutathione metabolism dysregulation^20,43^. However, the glucose use reprogramming and transport changes that occur in later ALD stages (i.e., severe AH) are likely to have downstream consequences and may result in feedback from other systemic metabolic dysregulation^5,7,44^.

We sought to further analyze how energy metabolism pathways were deregulated with ALD as many of these pathways (i.e., pentose phosphate pathway, glycolysis, TCA cycle, and OXPHOS) significantly correlated with ALD pseudotime. There is a tight balance between glycolysis and OXPHOS, both of which contribute to energy production, however OXPHOS serves as the primary energy source in the healthy liver^45,46^. This balance in energy production is severely diminished with alcohol consumption and disease onset, resulting in a switch in metabolic energy production, transitioning from the preferential energetic method of OXPHOS to glycolysis^47–49^. This metabolic switch is matched with an increase in glycolytic gene expression and metabolite levels as observed in Massey et al. 2021^8^. In this study, we found that all metabolic fluxes from glucose import to pyruvate production in the glycolysis pathway were upregulated in the severe AH patient cohort relative to the healthy control patients, most of which were significantly positively correlated with their calculated net enzymatic levels. Of interest, however, was the glucose import reaction (HMR_5029), as the metabolic fluxes were upregulated in patients with severe AH relative to healthy controls, which was in direct contrast to the calculated NELs. We discovered that one reaction downstream from glucose import, i.e., glucose to G6P production (HMR_4394), is encoded by various hexokinase genes, of which *Hkdc1* is the highest expressed. Interestingly, *Hkdc1* has been shown to be selectively upregulated with AH but not any other ALD or NALD^8^. The metabolic fluxes for glucose import and glucose to G6P production are significantly positively correlated, which facilitated our hypothesis that *Hkdc1* could be the main gene driving the upregulation of glucose import, resulting in the downstream upregulation of glycolytic fluxes in patients with AH.

In addition to characterizing the aberrant expression of *Hkdc1* in the livers of patients with AH, the role of *Hkdc1* in various other diseases, including gastric cancer and lung adenocarcinoma, has recently come into question^29,30^. For instance, the authors from Wang et al. 2023^30^ indicated that *Hkdc1* expression was upregulated in gastric cancer tissues relative to adjacent nontumor tissues and was associated with a worse prognosis. Further, they demonstrated that *Hkdc1* upregulation promoted glycolysis, cell proliferation, and tumorigenesis. Comparatively, Wang et al. 2020^29^ identified an upregulation of *Hkdc1* expression in lung adenocarcinoma samples, whose expression levels correlated with aberrant clinicopathological characteristics and resulted in the promotion of proliferation, migration, invasion, glycolysis, epithelial-to-mesenchymal transition, and tumorigenicity. The findings from these studies align with our observed pathogenic phenotype of elevated glycolytic flux in patients with severe AH.

In order to test our hypothesis that *Hkdc1* is the gene responsible for driving glucose use reprogramming and pyruvate remodeling, we performed in silico knockdown of *Hkdc1* in the severe AH and explant AH patient cohorts. Interestingly, we found three distinct subsets of patients based on their response to the knockdown: the Responsive patient cohort, which contained patients with glucose use fluxes that were significantly downregulated relative to the Refractory patient cohort, while patients in the Partially Responsive cohort had an intermediary response in rebalancing glycolytic metabolic fluxes in the direction of the healthy patient cohort. We explored the progressive, patient-specific response to intervention via *Hkdc1* knockdown by performing an unbiased differential expression analysis between the Responsive and Refractory patient cohorts to identify additional glycolytic genes potentially driving this patient-specific response. This analysis revealed a significant upregulation of *Hk1*, *Hk2*, and *Pkm* expression in the Refractory cohort relative to the Responsive cohort. We next performed combinatorial knockdown experiments for *Hkdc1*/*Hk1*, *Hkdc1*/*Hk2*, and *Hkdc1*/*Pkm* and found that only upon knockdown of both *Hkdc1* and *Pkm* did all patient groups produce a robust response, resulting in a significant downregulation of glucose use metabolic fluxes in the direction of the healthy patient cohort.

Similar to the behavior of *Hkdc1*, the expression of *Pkm* is detected at very low levels in the healthy liver but dramatically increases with liver disease^50–54^. Using quantitative phosphoproteomics collected from MASH rat model livers, one group identified that the activation of glycolysis in Kupffer cells during MASH was at least partially induced by the upregulation of *Pkm* via suppression of microRNA miR-122-5p^55^. In an additional study, *Pkm* was found to be upregulated in hepatocytes from mice with hepatic steatosis, and treatment with digoxin, a potent suppressor of *Pkm*-induced pathway activation, directly improved hepatocyte mitochondrial dysfunction and steatosis^56^. Lastly, in a study of ethanol-treated mice, it was shown that upregulated DRAM1, DNA damage-regulated modulator 1, increased the number of *Pkm*-enriched extracellular vesicles, thereby promoting macrophage activation and progression of ALD^57^.

To determine the extent of glucose use reprogramming of each patient prior to and following in silico knockdown intervention, we developed a linear SVM regression model that was trained using the HMR_5029, HMR_4394, and HMR_4381 metabolic fluxes as features. The resulting regression model could then calculate the extent of glucose use reprogramming and could predict the patient-specific progressive responsiveness to intervention via *Hkdc1* knockdown alone and the combinatorial knockdown of *Hkdc1* and *Pkm*. To test the functionality of this scoring method, we used a publicly available bulk RNAseq dataset consisting of 13 liver samples from patients with severe AH^34^. We found that prior to KD, metabolic fluxomes of the severe AH patients in the validation cohort clustered closely with the severe AH patients in the Responsive and Partially Responsive groups from the original cohort. We then used the SVM model to test the glucose use reprogramming scores of these patients in comparison to our original cohort of patients. In accordance with the glucose use reprogramming scores of severe AH patients in the Responsive group of the original cohort, we found that all 13 severe AH patients in the validation cohort exhibited significant downregulation of glucose use reprogramming scores following *Hkdc1* knockdown alone and in combination with *Pkm* that were in range with the healthy control patients.

Glycolytic flux is mainly controlled by several rate-limiting enzymes, including hexokinases and pyruvate kinases^30,58–60^. In our patient-specific metabolic modeling study, we experimented with in silico knockdown of these critical enzymes. Following in silico knockdown of *Hkdc1* alone, we identified that patients with AH exist along a response continuum, with some patients rebalancing their glycolytic fluxes more robustly in the direction of healthy patients. Individuals who did not respond as robustly to *Hkdc1* knockdown alone may require a combination therapy of *Hkdc1* and *Pkm* to significantly shift the glycolytic fluxes of the previously less responsive patient group towards the healthy phenotype. Through the use of a glucose use reprogramming score, we were able to predict the extent to which a patient with severe AH may respond to a gene knockdown intervention based on their upstream glucose import and glycolytic metabolic fluxes. Through clinical deployment of the glucose use reprogramming scoring methodology, physicians can be better informed on the metabolic state of a patient’s liver. Further, we propose that targeting specific metabolites within the glycolysis pathway based on the patient’s predicted response to therapies may serve as a potential therapeutic intervention for individuals with AH. Thus, the current standard of care treatment for patients with AH can be improved by developing individualized therapeutics that target metabolites in the glycolysis pathway based on a patient’s predicted response to treatment.

This study is the first of its kind to generate personalized metabolic models for patients across the ALD spectrum. However, there are limitations to this modeling effort that should be acknowledged, specifically pertaining to sample sizes, collected patient information, data integration techniques, sensitivity of the modeling approach, and the uncertainty in FBA prediction. Firstly, the study is limited by the number of patients involved in the study as well as the information collected from the patients at the time of biopsy. For instance, metabolic processes are quite sensitive to timing (i.e., the circadian clock), perturbations (i.e., alcohol intake), and fed vs. fasting status^61–64^. Therefore, a limitation of the present modeling methodology is the absence of patient-level information regarding fasting status, alcohol use level at the time of biopsy, and the timing of biopsy collection. Furthermore, while all patients recruited in the clinical trial must have been active drinkers ( > 40 grams of alcohol/day for females, >60 grams of alcohol/day for males for 6 months or more, with less than 60 days of abstinence prior to the onset of jaundice; dbGaP accession number phs001807.v1.p1), the amount of alcohol consumed may differ. This may result in slight metabolic flux changes in drinking patients with early ALD when the disease is still reversible and ethanol substrates are more freely available in patients consuming more alcohol. However, we postulate that the patterns observed in patients with severe ALD would remain relatively constant, as the disease and the damage to the liver is irreversible.

Multiple methods exist for integrating omics data into the metabolic modeling pipeline such as at the structural network level, where pruning-based model extraction methods yield context-specific GEMs^65^. However, for the purpose of this study, we chose to integrate the transcriptomics data during FBA prediction using Pheflux^22^ to generate personalized metabolic fluxomes. Further, because Pheflux does not require an optimization function, it is quite sensitive to the transcriptomics data provided. Lastly, there is uncertainty in FBA prediction as the algorithm assumes the system is at steady state i.e., the rate of the change of the metabolites over time is zero, which is biologically imperfect as even homogeneous cell populations may exhibit heterogeneity in uptake, secretion, and growth rates^66^. Despite these limitations, the approach presented here remains a powerful tool for developing personalized, data-informed metabolic fluxomes. These limitations can be overcome through advancements in metabolic modeling approaches and flux balance analysis techniques, thus enabling broader application across various scientific domains.

## Methods

### Human liver bulk RNAseq data

A total of 90 human liver biopsy samples were obtained as per the “Human Biorepository Core from the NIH-funded international InTeam consortium” (7U01AA021908-05). The 90 patient samples are stratified into seven distinct disease states and a healthy control state: explant alcoholic hepatitis (explant AH, *n* = 10), severe alcoholic hepatitis (severe AH, *n* = 18), non-severe alcoholic hepatitis (non-severe AH, *n* = 11), early alcoholic steatohepatitis (early ASH, *n* = 12), healthy (*n* = 10), compensated cirrhosis (comp. cirrhosis, *n* = 9), hepatitis C virus (HCV, *n* = 10), and metabolic dysfunction-associated steatohepatitis (MASH, *n* = 9). Patients had a median age of 45 in the explant AH disease state category, 51 in severe AH, 46 in non-severe AH, 52 in early ASH, 32 in the healthy controls, 61 in compensated cirrhosis, 46 in HCV, and 49 in MASH.

Samples classified as explant AH originated from patients undergoing a liver transplant. At the time of transplant, the liver was removed from the body and was identified as having AH. A biopsy sample was then taken from the explanted liver. Samples exhibiting severe AH were clinically and pathology-proven AH and had a MELD score > 21. The non-severe AH patients were heterogeneous in that some displayed AH criteria (criteria discussed above) while some did not, but all had a MELD score < 21. The early ASH patients were non-obese, actively misused alcohol, and displayed liver damage (elevation of liver enzymes) but did not have liver dysfunction (i.e., asymptomatic, normal bilirubin, normal prothrombin levels). They also presented mild elevation of transaminases and histologic criteria of ASH but never had AH episodes. The control samples were taken before any clamping or ischemia from the normal liver surrounding apparent liver nodules as a result of metastases. The patient samples from the compensated cirrhosis, HCV, and MASH cohorts were all clinically and pathology-proven proven, and none of the patients had any AH episodes. Patients from the compensated cirrhosis cohort were asymptomatic and had HCV and cirrhosis but did not have ascites, variceal hemorrhage, hepatic encephalopathy, or jaundice. Patients within the HCV cohort did not have cirrhosis, and patients within the MASH cohort were defined according to Keiner’s Criteria and displayed accumulated hepatic fat histologically and had above-average body mass index (BMI > 25).

Patient inclusion and exclusion criteria in the clinical trial, as well as additional information about the samples, can be found on the clinical trial website (https://clinicaltrials.gov/study/NCT02075918).

### RNAseq data normalization and outlier detection

The raw count’s matrix was counts-per-million (CPM) normalized, and patient samples were analyzed for plausible outliers. One explant AH patient sample was identified as an outlier (greater than six standard deviations above the mean) in the principal component analysis (PCA) plot and was removed from the downstream analysis, resulting in a total of 89 patient samples (Supplementary Figs. 1A, B). The prcomp RStudio package was utilized for calculating the principal components and ggplot2 was used for plotting.

### Reference Human1 metabolic model

The Human1 metabolic model was used for downstream metabolic flux prediction^21^. The Human1 model was utilized as it represents a consensus genome-scale metabolic model (GEM) that was developed following extensive integration and curation from previous human metabolic models. Briefly, the Human1 model consists of 13,416 reactions, 8458 metabolites, 3,628 genes, 9 cellular compartments, and 143 biological subsystems^21^. The shorthand nomenclature for each cellular compartment is as follows: cytosol (c), extracellular (e), peroxisome (x), golgi apparatus (g), lysosome (l), endoplasmic reticulum (r), nucleus (n), mitochondria (m), inner mitochondria (i). Each of the reactions is represented by a gene-protein rule (GPR), which describes the gene-encoding enzymes, isozymes, and complex subunits. Genes separated by OR encode isozymes, while those separated by AND encode complex subunits. Therefore, one isozyme can compensate for the deletion of an associated isozyme, however, deletion of a complex subunit inactivates the entire enzyme complex such that the reaction activity cannot be maintained. The details of each metabolic subsystem and reaction, including the equation, GPR, compartment, etc., can be found at https://metabolicatlas.org/.

### Patient-specific metabolic fluxome predictions

Patient-specific metabolic fluxomes were predicted using the Pheflux algorithm^22^. This methodology seeks to infer the most likely and least biased fluxome by maximizing Shannon’s entropy of fluxes per mRNA without the need for an objective function. Maximization of Shannon’s entropy is equivalent to minimizing the forward Kullback-Leiber divergence between the fluxome and transcriptome. The input to the Pheflux algorithm is the CPM-normalized RNAseq data and a reference metabolic network, for which we used the Human1 network^21^. The output is the patient-specific metabolic fluxomes, one flux for each reaction in the Human1 network. The Pheflux algorithm was implemented in Python using the source code found in the author’s GitHub repository: https://github.com/mrivas/pheflux.

Nonzero fluxes were identified as those greater than zero for all patients for a given reaction. An empirical cumulative distribution function (ecdf) plot was generated for the nonzero log10-transformed metabolic fluxes, and the knee point was established as the cutoff for reactions with significant flux values (Supplementary Fig. 1c). The coordinates of the knee point are (0.001, 0.534), corresponding to a flux cutoff at 0.001 mmol/gDW/g, which consists of 53.4% of the entire fluxome. The ecdf function from the stats package in RStudio was utilized to calculate the ecdf, and ggplot2 was utilized for plotting.

### Calculating Net Enzymatic Levels (NEL)

We define the Net Enzymatic Level (NEL) as the GPR calculation for each reaction in the Human1 metabolic network using the associated gene expression data. According to the Pheflux algorithm, the gene expression is added for genes separated by OR and the minimum expression between genes is taken when separated by AND.

### In silico Gene Knockdown Experiments

For each gene knockdown experiment, a scaling factor was calculated by dividing the maximum gene expression of the healthy patients by the maximum gene expression of the severe AH patients for a given gene. The original gene expression for the severe AH patients was then multiplied by the gene-specific scaling factor to achieve gene expression for severe AH patients within the range of the healthy patients. For simplicity, we consider a 50% knockdown to be ½ of the original severe AH gene expression for a given gene knockdown. The entire metabolic transcriptome with the new gene knockdown values for the severe AH patients is then utilized as input to the Pheflux algorithm for metabolic flux prediction in the same manner as that of the original cohort.

### Liver metabolomics

Publicly available liver metabolomics data from patients with AH (*n* = 15) and healthy controls (*n* = 12) were obtained from Massey et al. 2021^8^. The normalized, rescaled metabolite levels were used for downstream analysis. Statistical significance was determined by Wilcoxon Rank Sum testing.

### Linear Support Vector Machine (SVM) regression model for calculating glucose use reprogramming scores

The following features (fluxes) were considered for building a linear SVM regression model of glucose use reprogramming: HMR_5029, HMR_4394, HMR_4381, HMR_4379, HMR_4375, HMR_4391, HMR_4373, HMR_4368, HMR_4365, HMR_4363, HMR_4358. The training dataset consisted of these fluxes for healthy patients and those with ALD (early ASH, nonsevere AH, severe AH, and explant AH). We considered the patients in the explant AH cohort as belonging to the severe AH cohort, as these patients had severe AH but required a liver transplant. We trained the machine learning model on the disease progression from healthy (score = 1, N = 10), to early ASH (score = 2, N = 12), to nonsevere AH (score = 3, N = 11), and severe AH (score = 4, N = 28). We utilized a recursive feature elimination (RFE) algorithm with leave-one-out cross-validation (LOOCV) to identify the most pertinent predictors of disease state progression. We performed this feature selection in RStudio using the rfe and rfeControl functions within the caret package. We iterated through the rfe algorithm using 1 to 11 features to experiment with the resulting model’s predictive capabilities for between 1 and 11 of the glucose use fluxes. For each iteration, we trained a linear SVM model on the selected features from the rfe algorithm. We then tested the resulting model on the trained data and on our testing dataset, which consisted of the fluxes from the severe AH and explant AH cohorts after in silico knockdown of *Hkdc1* and *Hkdc1*/*Pkm* double knockdown. We chose the features of the linear SVM model based on the following criteria: 1) scores with the highest correlation with ALD pseudotime, 2) scores with the largest significant difference between Group 1 and Group 3 following *Hkdc1* knockdown, 3) scores with the largest significant difference between the severe AH patient cohort without knockdown and Group 3 following *Hkdc1*/*Pkm* double knockdown, and 4) scores with the largest significant difference following Hkdc1 knockdown vs. *Hkdc1*/*Pkm* double knockdown. The resulting linear SVM regression model included HMR_5029, HMR_4394, and HMR_4381 fluxes as the features.

### Glucose use reprogramming score in the validation dataset

We utilized a publicly available bulk RNAseq dataset that contained 13 patients with severe AH^34^ (GSE143318). The raw data was downloaded from GEO under accession number GSE143318 and CPM-normalized. The resulting CPM-normalized gene expression data was input to the Pheflux algorithm along with the Human1 metabolic network to generate a total of 13 patient-specific metabolic fluxomes. Then, *Hkdc1* knockdown and *Hkdc1*/*Pkm* double knockdown experiments were performed. The final linear SVM regression model, which was trained on the progression of ALD and had HMR_5029, HMR_4394, and HMR_4381 fluxes as the features, was utilized to predict the glucose use reprogramming scores for the patients in the validation cohort with and without gene knockdowns.

### Constructing a decision tree for predicting patient-specific glucose use phenotypes

A decision tree was constructed using the most dominant gene expression from each of the main glycolysis reactions from glucose import (HMR_5029) to pyruvate production (HMR_4358). These genes included *Slc2a2, Hkdc1, Hk1, Gpi, Pfkl, Pfkp, Aldob, Tpi1, Gapdh, Pgk1, Pgam1, Eno1, Pkm*, and *Pklr*. The *rpart*, and *rpart.plot* packages in RStudio were utilized to generate and plot the decision tree, respectively. Because our sample size of 18 severe AH and explant AH patients is relatively low, we set the minsplit and minbucket parameters in the rpart.control function equal to 2. Following the generation of the decision tree, a confusion matrix was generated, and the accuracy of the model was calculated using the *caret* package in RStudio.

### Dimensionality Reduction and Pseudotime Analysis

Uniform Manifold Approximation and Projection (UMAP) dimensionality reduction was performed using the *umap* package in RStudio. Principal component analysis (PCA) was performed using the *dataVisEasy* package in RStudio. Pseudotemporal progression analyses were performed on the dimensionality reduction data (UMAP or PCA) in RStudio using the *slingshot* and *slingPseudotime* functions within the slingshot package.

### ALD trajectory pseudotime

For the healthy patients and those with ALD (early ASH, nonsevere AH, severe AH, explant AH), we used the UMAP coordinates of the plot for metabolic genes alone from the Human1 metabolic network. The UMAP coordinates and patient sample annotations were input to the slingshot function in RStudio with the start.clus parameter set to the healthy patient cohort and the end.clus parameter set to the explant AH patient cohort.

### Pseudotemporal progression of patients with severe AH (including explant AH) using glucose utilization metabolic fluxes

We considered the fluxes corresponding to the following reactions: HMR_5029, HMR_4394, HMR_4381, HMR_4379, HMR_4375, HMR_4391, HMR_4373, HMR_4368, HMR_4365, HMR_4363, and HMR_4358 for severe patients in Groups 1-3. Pseudotimes were calculated separately for severe AH patient samples before any gene knockdown and following in silico *Hkdc1* knockdown by inputting the corresponding PCA coordinates into the slingshot function without providing cluster labels.

### Data Manipulation, Clustering, Statistical Testing, and Plotting

The *kmeans* function in the stats package in RStudio was utilized for k-means clustering of metabolic fluxes. A Sankey diagram was generated to show the flow of patient samples from k-means clustering of fluxes at two levels of resolution. The *sankeyNetwork* function within the *networkD3* package in RStudio was used for visualization of the Sankey diagram.

Metabolic fluxes greater than 0.001 mmol/gDW/g underwent hierarchical clustering, resulting in groups of reactions that exhibited similar metabolic flux behavior. For each of the groups, the subsystems were tabulated, and overrepresentation analyses were performed. Specifically, if the subsystem is present more than expected in a single group, the overrepresentation analysis will yield a significant *p*-value (*p* < 0.05).

One-sample Student’s paired t-testing was used to determine the significance between severe AH patient fluxes with vs. without gene knockdowns, while one-sample Student’s unpaired t-testing was used to determine the significance between all other groups. The t.test function in the stats package in RStudio was used for t-testing with the paired parameter set to True for paired t-testing and False for unpaired t-testing. We consider a *p*-value < 0.05 to be statistically significant.

### Statistics and Reproducibility

The 90 patient samples from the original cohort (7U01AA021908-05) were stratified into seven disease states (explant AH: *n* = 10, severe AH: *n* = 18, non-severe AH: *n* = 11, early ASH: *n* = 12, comp. cirrhosis: *n* = 9, HCV: *n* = 10, MASH: *n* = 9) and a healthy control state (*n* = 10) based on the inclusion/exclusion criteria from the clinical trial (https://clinicaltrials.gov/study/NCT02075918). One explant AH outlier sample was removed from downstream data analysis as it was greater than six standard deviations above the mean. For overrepresentation analyses, Fisher’s Exact Test was used to calculate odds ratios and p-values. For all other statistical testing, a one-sample Student’s t-test (paired and unpaired) was used to determine significance. All significant p-values and the statistical tests used are indicated in the figures and text. Statistics were estimated and plots were generated using RStudio (v4.2.2).

### Ethics statement

This study used published de-identified patient data. Hence, an institutional review board assessment was not required for the present study. The original study that developed the dataset was conducted in accordance with the Declaration of Helsinki and approved by the applicable Institutional Review Board. Informed consent was obtained from all subjects involved in the study. The details of the published data are provided in the Materials and Methods section.

### Reporting summary

Further information on research design is available in the Nature Portfolio Reporting Summary linked to this article.

## Supplementary information

Transparent Peer Review file

Supplementary Information

Reporting Summary

## Data Availability

The raw RNAseq data used in this study are openly available in the Database of Genotypes and Phenotypes (dbGAP) of the National Center for Biotechnology Information (United States National Library of Medicine, Bethesda, MD) under accession number phs001807.v1.p1. In order to validate our method of glucose use reprogramming scoring, we utilized a publicly available, independent bulk RNAseq dataset consisting of 13 liver samples from patients with severe AH (GSE143318). All source data and code necessary for reproducing and replicating the modeling studies and figures are available via GitHub at https://github.com/Daniel-Baugh-Institute/Patient-specific_AH_LiverGEMStudy and via Zenodo (https://doi.org/10.5281/zenodo.19472115)^67^.

## Code availability

The code for reproducing and replicating the modeling studies and figures are available via GitHub at https://github.com/Daniel-Baugh-Institute/Patient-specific_AH_LiverGEMStudy and via Zenodo (https://doi.org/10.5281/zenodo.19472115)^67^.
