# Supplementary material for: Patient-specific modeling identifies metabolic interventions for reversing glucose use reprogramming in alcohol-associated hepatitis
Source: Commun Biol. 2026 Jun 4;9:1181. doi: 10.1038/s42003-026-10407-5 (PMC13554195; doi:10.1038/s42003-026-10407-5)
Supplement: Supplementary file 1 [file 42003_2026_10407_MOESM1_ESM.pdf]

nature portfolio

Peer Review File

Patient-specific modeling identifies metabolic interventions for reversing glucose use reprogramming in alcohol-associated hepatitis

Corresponding Author: Professor Rajanikanth Vadigepalli

This file contains all reviewer reports in order by version, followed by all author rebuttals in order by version.

Version 0:

Reviewer comments:

Reviewer #1

(Remarks to the Author)
This paper performs an in-depth analysis of the metabolic fluxes in patients affected with ALD (increasing severities) and compares these with healthy controls and other liver diseases not caused by alcohol. The study demonstrates a progressive reprogramming of glucose metabolism in AH using genome-scale metabolic models integrated with transcriptomic/metabolomic data and identifies two genes (HKDC1 and PKM) as drivers of the altered metabolism. The authors perform an in silico knock-down of genes associated with glucose metabolism and demonstrate that targeting HKDC1 and PKM can rebalance the glycolytic fluxes toward a healthy phenotype. Furthermore, they develop a machine-learning derived glucose use reprogramming score to assess disease severity and predict patient-specific response to the knocking down of these genes. Finally, they use an independent dataset to validate their model and scoring system.

The study is very innovative, robust and well written in a way that is accessible to non-experts on computational or system biology. In particular, I found very novel and with great potential the use of in silico gene knockdown to stratify patients into therapeutically responsive subgroups and the combination of this with a machine learning-derived score to predict intervention outcomes. The integration of patient-specific GEMs with transcriptomics and metabolomics data to identify metabolic intervention points in AH is highly innovative. The findings are highly relevant to the liver disease and systems biology communities. The identification of HKDC1 and PKM as therapeutic targets could influence future drug development or biomarker discovery in AH and potentially other metabolic liver diseases.

In the Discussion, the authors cite published data supporting their findings, especially the role of PKM in metabolism in the context of different diseases, which helps justify the absence of direct experimental validation in their study. Finally, the authors focus on the limitations of their study to the modelling, but I would recommend that other points should be discussed:

The lack of information on fasting status, alcohol use at the time of biopsy, and time of day is a significant limitation for a study focused on metabolism. I appreciate this would not be feasible for these datasets, but I would strongly suggest acknowledging these limitations in the Discussion or including a brief sensitivity analysis or rationale explaining why the modelling approach is still valid despite these confounders.

Considering that some patients are current drinkers while others are abstinent, I would recommend commenting on how alcohol metabolism could affect the metabolic fluxes and therefore impact the substrate availability to the described metabolic pathways.

Dr Elena Palma
Principal Investigator & Lecturer (adj.)
Roger Williams Institute of Liver Studies
School of Immunology & Microbial Sciences, Faculty of Life Sciences and Medicine
King’s College London, Foundation for Liver Research and King’s College Hospital
111 Coldharbour Lane, London, SE5 9NT

Reviewer #2

(Remarks to the Author)
The Patient-1 specific modeling identifies metabolic interventions for reversing glucose use reprogramming in alcohol-associated hepatitis is a genome scale analysis of 89 patient samples with AH and integrating with transcriptomics from AH patients. The manuscript highlights the metabolic pathways that are dysregulate in AH and show that knockdown of two glycolytic genes, Hkdc1 and Pkm, can rebalance the metabolic fluxes toward a healthy liver metabolic phenotype. The manuscript highlights the power of big data analysis and integrating data to identify global changes and at the same time identify single genes that can be targeted for therapeutic strategies.
Please comment on whether the change in these global patterns are the cause or consequence of the disease.
Please comment/discuss on or postulate why fatty acid/cholesterol synthesis is increased with alcohol. The potential mechanisms.
The data suggest that there is a decrease in OXPHOS, does this also contribute significantly to the energy deficit similar to or more than glycolysis.
Minor comments
Please change terminology to reflect the current nomenclature - NASH,
Line 145- Rephrase"oppositely"

Reviewer #3

(Remarks to the Author)
I have expertise in computer science, and I can comment on the computational aspects of the paper.

The analysis done by the authors looks fine. However, the number of samples is quite small. The methods used in the paper are standard methods, but I can see the authors have not claimed novelty of the algorithm.

I would suggest improving the quality of the GitHub repository with detailed README for reproducibility of the method and to make it easier for researchers to build on this research. Add appropriate docstring to explain functions. The jupyter notebooks look fine. However, list of requirements for the python environment are missing.

Version 1:

Reviewer comments:

Reviewer #2

(Remarks to the Author)
The comments are satisfactorily addressed and has strenghtened the manuscript

Reviewer #3

(Remarks to the Author)
I do not see any changes to the README file in the repo https://github.com/Daniel-Baugh-Institute/Patient-specific_AH_LiverGEMStudy. I would suggest adding a requirements file instead of information.txt. Here is a blogpost I found on the internet which may help https://learnpython.com/blog/python-requirements-file/. The authors can then update the Jupyter notebook with a `pip install -r requirments.txt` cell. Documentation of the code is the same as before which was update about an year ago.

Version 2:

Reviewer comments:

Reviewer #3

(Remarks to the Author)
The authors have updated the GitHub repo documentation. I have no further comments.

Open Access This Peer Review File is licensed under a Creative Commons Attribution 4.0 International License, which permits use, sharing, adaptation, distribution and reproduction in any medium or format, as long as you give appropriate credit to the original author(s) and the source, provide a link to the Creative Commons license, and indicate if changes were made.
In cases where reviewers are anonymous, credit should be given to 'Anonymous Referee' and the source.
The images or other third party material in this Peer Review File are included in the article's Creative Commons license, unless indicated otherwise in a credit line to the material. If material is not included in the article's Creative Commons license and your intended use is not permitted by statutory regulation or exceeds the permitted use, you will need to obtain permission directly from the copyright holder.
To view a copy of this license, visit https://creativecommons.org/licenses/by/4.0/

Response to Reviewers:

Reviewer #1:

This paper performs an in-depth analysis of the metabolic fluxes in patients affected with ALD (increasing severities) and compares these with healthy controls and other liver diseases not caused by alcohol. The study demonstrates a progressive reprogramming of glucose metabolism in AH using genome-scale metabolic models integrated with transcriptomic/metabolomic data and identifies two genes (HKDC1 and PKM) as drivers of the altered metabolism. The authors perform an in silico knock-down of genes associated with glucose metabolism and demonstrate that targeting HKDC1 and PKM can rebalance the glycolytic fluxes toward a healthy phenotype. Furthermore, they develop a machine-learning derived glucose use reprogramming score to assess disease severity and predict patient-specific response to the knocking down of these genes. Finally, they use an independent dataset to validate their model and scoring system.

The study is very innovative, robust and well written in a way that is accessible to non-experts on computational or system biology. In particular, I found very novel and with great potential the use of in silico gene knockdown to stratify patients into therapeutically responsive subgroups and the combination of this with a machine learning-derived score to predict intervention outcomes. The integration of patient-specific GEMs with transcriptomics and metabolomics data to identify metabolic intervention points in AH is highly innovative. The findings are highly relevant to the liver disease and systems biology communities. The identification of HKDC1 and PKM as therapeutic targets could influence future drug development or biomarker discovery in AH and potentially other metabolic liver diseases.

In the Discussion, the authors cite published data supporting their findings, especially the role of PKM in metabolism in the context of different diseases, which helps justify the absence of direct experimental validation in their study. Finally, the authors focus on the limitations of their study to the modelling, but I would recommend that other points should be discussed:

The lack of information on fasting status, alcohol use at the time of biopsy, and time of day is a significant limitation for a study focused on metabolism. I appreciate this would not be feasible for these datasets, but I would strongly suggest acknowledging these limitations in the Discussion or including a brief sensitivity analysis or rationale explaining why the modelling approach is still valid despite these confounders.

Considering that some patients are current drinkers while others are abstinent, I would recommend commenting on how alcohol metabolism could affect the metabolic fluxes and therefore impact the substrate availability to the described metabolic pathways.

Dr Elena Palma
Principal Investigator & Lecturer (adj.)
Roger Williams Institute of Liver Studies
School of Immunology & Microbial Sciences, Faculty of Life Sciences and Medicine
King's College London, Foundation for Liver Research and King's College Hospital
111 Coldharbour Lane, London, SE5 9NT

Response: We thank the reviewer for their comments and appreciate the support. We agree that these limitations should be explicitly stated and have included these points in the revised version of the manuscript. We have included an explanation of the limitations mentioned by the reviewer in the "Limitations of the study" section of the manuscript (beginning on Line 684). Specifically, on Lines 689 to 702, we discuss the limitations of the dataset including the number of patients enrolled in the study as well as the lack of patient-level information on fasting status, alcohol use at the time of biopsy, and time of day of biopsy. Additionally, we postulate on how the amount of alcohol consumed may affect the metabolic fluxes and impact the substrate availability. Specifically, in Lines 689 to 702 we state, "Firstly, the study is limited by the number of patients involved in the study as well as the information collected from the patients at the time of biopsy. For instance, metabolic processes are quite sensitive to timing (i.e., the circadian clock), perturbations (i.e., alcohol intake), and fed vs. fasting status 61–64. Therefore, a limitation of the present modeling methodology is the absence of patient-level information regarding fasting status, alcohol use level at the time of biopsy, and the timing of biopsy collection. Furthermore, while all patients recruited in the clinical trial must have been active drinkers (>40 grams of alcohol/day for females, >60 grams of alcohol/day for males for 6 months or more, with less than 60 days of abstinence prior to the onset of jaundice; dbGaP accession number phs001807.v1.p1), the amount of alcohol consumed may differ. This may result in slight metabolic flux changes in drinking patients with early ALD when the disease is still reversible and ethanol substrates are more freely available in patients consuming more alcohol. However, we postulate that the patterns observed in patients with severe ALD would remain relatively constant, as the disease and the damage to the liver is irreversible."

Reviewer #2:

The Patient-1 specific modeling identifies metabolic interventions for reversing glucose use reprogramming in alcohol-associated hepatitis is a genome scale analysis of 89 patient samples with AH and integrating with transcriptomics from AH patients. The manuscript highlights the metabolic pathways that are dysregulate in AH and show that knockdown of two glycolytic genes, Hkdc1 and Pkm, can rebalance the metabolic fluxes toward a healthy liver metabolic phenotype. The manuscript highlights the power of big data analysis and integrating data to identify global changes and at the same time identify single genes that can be targeted for therapeutic strategies.

Response: We thank the reviewer for their commentary and feedback and have updated the manuscript to reflect the reviewer's suggestions.

Comment 1: Please comment on whether the change in these global patterns are the cause or consequence of the disease.

Response: While the reviewer makes a good point that it would be very beneficial to know whether the global patterns observed with ALD progression are causal or consequential, it is very difficult to parse out this information given the relatively small size of the patient cohort and the type of data collected. We have added this point to the discussion in Lines 574 – 585, in which we state "However, the global patterns that correlate with ALD progression do not readily separate into causal vs. consequential as the metabolic changes observed are systemic, affecting other organs and biological processes in the body. Additionally, there is feedback to the liver resulting in further disease progression. Therefore, trying to parse out these causal vs. consequential effects in the data is quite difficult without additional perturbation information or a substantial increase in the patient cohort size, thereby including naturally occurring perturbations. Despite this caveat, some metabolic shifts that occur in the less severe disease states (i.e., early ASH) are more likely to be causative based on known biology of ALD, such as that of glutathione metabolism dysregulation [20,43]. However, the glucose use reprogramming and transport changes that occur in later ALD stages (i.e., severe AH) are likely to have downstream consequences and may result in feedback from other systemic metabolic dysregulation [5,7,44]."

Comment 2: Please comment/discuss on or postulate why fatty acid/cholesterol synthesis is increased with alcohol. The potential mechanisms.

Response: We have included additional text in the discussion to explain the biological mechanism of increased fatty acid metabolism and altered cholesterol metabolism

associated with ALD. Specifically, in Lines 564 – 568, we state “This increase in fatty acid metabolism with ALD has been observed in both human and mouse studies, the mechanism of which is due to the combinatorial effect of increased glycerolipid synthesis, decreased fatty acid oxidation, and alteration of fatty acid export processes [35–38]. Chronic alcohol consumption has also been shown to disrupt cholesterol metabolism leading to ALD progression [39–41].”

Comment 3: The data suggest that there is a decrease in OXPHOS, does this also contribute significantly to the energy deficit similar to or more than glycolysis.

Response: This is an important point made by the reviewer and we have addressed this further in the discussion section of the revised version of the manuscript. Specifically, in Lines 588 – 594, we state, “There is a tight balance between glycolysis and OXPHOS, both of which contribute to energy production, however OXPHOS serves as the primary energy source in the healthy liver [45,46]. This balance in energy production is severely diminished with alcohol consumption and disease onset, resulting in a switch in metabolic energy production, transitioning from the preferential energetic method of OXPHOS to glycolysis [47–49]. This metabolic switch is matched with an increase in glycolytic gene expression and metabolite levels as observed in Massey et al. 2021 [8].”

Minor comments:
Please change terminology to reflect the current nomenclature - NASH,
Line 145- Rephrase"oppositely"

Response: We have updated the main text, figures, and supplementary material to reflect the new nomenclature for NASH, namely MASH. We have also rephrased “oppositely” in Line 145 by combining this sentence with the previous one to read “Additionally, nearly all of the early ASH patient samples (91.7%), as well as roughly half of the nonsevere AH patient samples (54.5%), were in Cluster 1, while Cluster 2 contains most of the patients with extreme cases of ALD (94.4% of severe AH and 70% of explant AH patient samples).” (Lines 154-157 in revised version of the manuscript).

Reviewer #3:

This reviewer has expertise in computer science, and can comment on the computational aspects of the paper.

The analysis done by the authors looks fine. However, the number of samples is quite small. The methods used in the paper are standard methods, but I can see the authors have not claimed novelty of the algorithm.

I would suggest improving the quality of the GitHub repository with detailed README for reproducibility of the method and to make it easier for researchers to build on this research. Add appropriate docstring to explain functions. The jupyter notebooks look fine. However, list of requirements for the python environment are missing.

Response: We thank the reviewer for extensively reviewing the code associated with the manuscript and quality of the GitHub repository. We have now included additional information in the README file for reproducing the analyses and figures present in the manuscript. We have also ensured that files are readily available for installing Python and RStudio dependencies for replicating the coding environments.

Response to Reviewer Comments

To the Editor:
We have addressed a minor edit in the Github README as below. We also updated the affiliation information of corresponding author.

Reviewer #1:
The comments have been satisfactorily addressed.

Response: Thank you.

Reviewer #2:
The comments are satisfactorily addressed and has strengthened the manuscript.

Response: Thank you.

Reviewer #3:
I do not see any changes to the README file in the repo https://github.com/Daniel-Baugh-Institute/Patient-specific_AH_LiverGEMStudy. I would suggest adding a requirements file instead of information.txt. Here is a blogpost I found on the internet which may help https://learnpython.com/blog/python-requirements-file/. The authors can then update the Jupyter notebook with a `pip install -r requirments.txt` cell. Documentation of the code is the same as before which was update about an year ago.

Response: We thank the reviewer for their comments and have updated the GitHub README file to include additional information for reproducing the analyses and figures in the manuscript. Specifically, we suggest creating a conda environment with the specific version of python used for replication. Additionally, we have now included a "requirements.txt" file instead of an "information.txt" file such that all python dependencies can be installed using the `pip install -r requirements.txt`command. The updated GitHub repository is publicly available at https://github.com/Daniel-Baugh-Institute/Patient-specific_AH_LiverGEMStudy.
