# Supplementary material for: Patient-specific modeling identifies metabolic interventions for reversing glucose use reprogramming in alcohol-associated hepatitis
Source: Commun Biol. 2026 Jun 4;9:1181. doi: 10.1038/s42003-026-10407-5 (PMC13554195; doi:10.1038/s42003-026-10407-5)
Supplement: Supplementary file 2 [file 42003_2026_10407_MOESM2_ESM.pdf]

# Supplementary Information

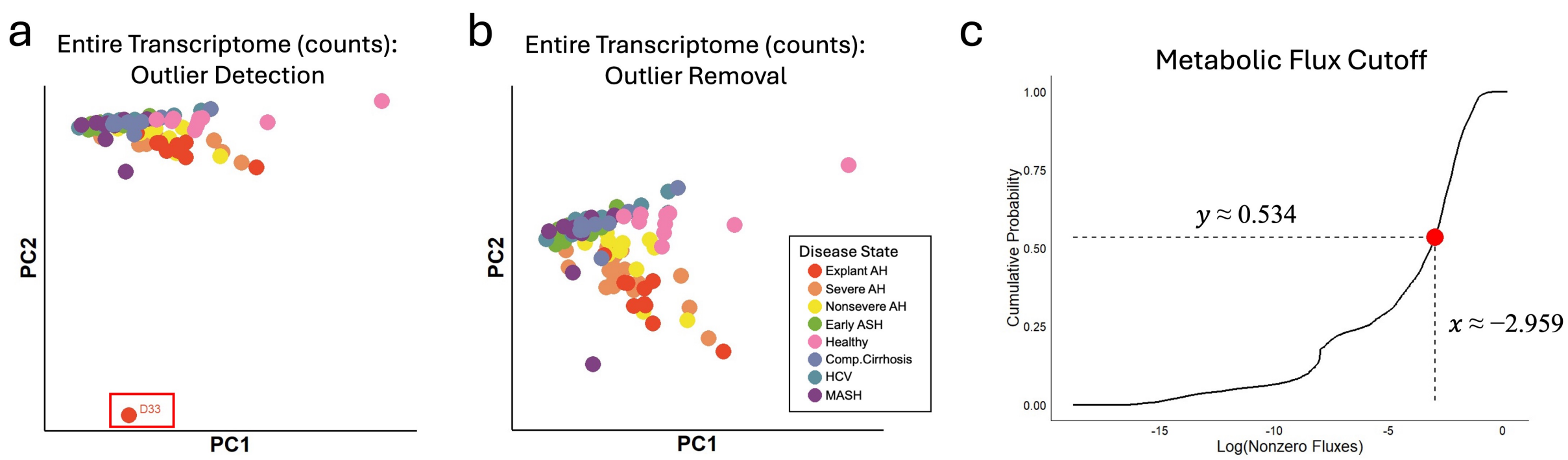


**Supplementary Fig. 1**. **Transcriptomic outlier detection and setting metabolic flux cutoff.** (**a**) PCA plot of the entire transcriptome counts for all patients with sample D33 (explant AH) identified as an outlier. (**b**) PCA plot of the entire transcriptome counts for all patients with outlier sample D33 (explant AH) removed. (**c**) ECDF plot shown for all nonzero log10-transformed metabolic fluxes. The knee point was identified as (-2.958, 0.534), so the metabolic flux cutoff was set at 10^(-2.958) ≈ 0.001 mmol/gDW/g.

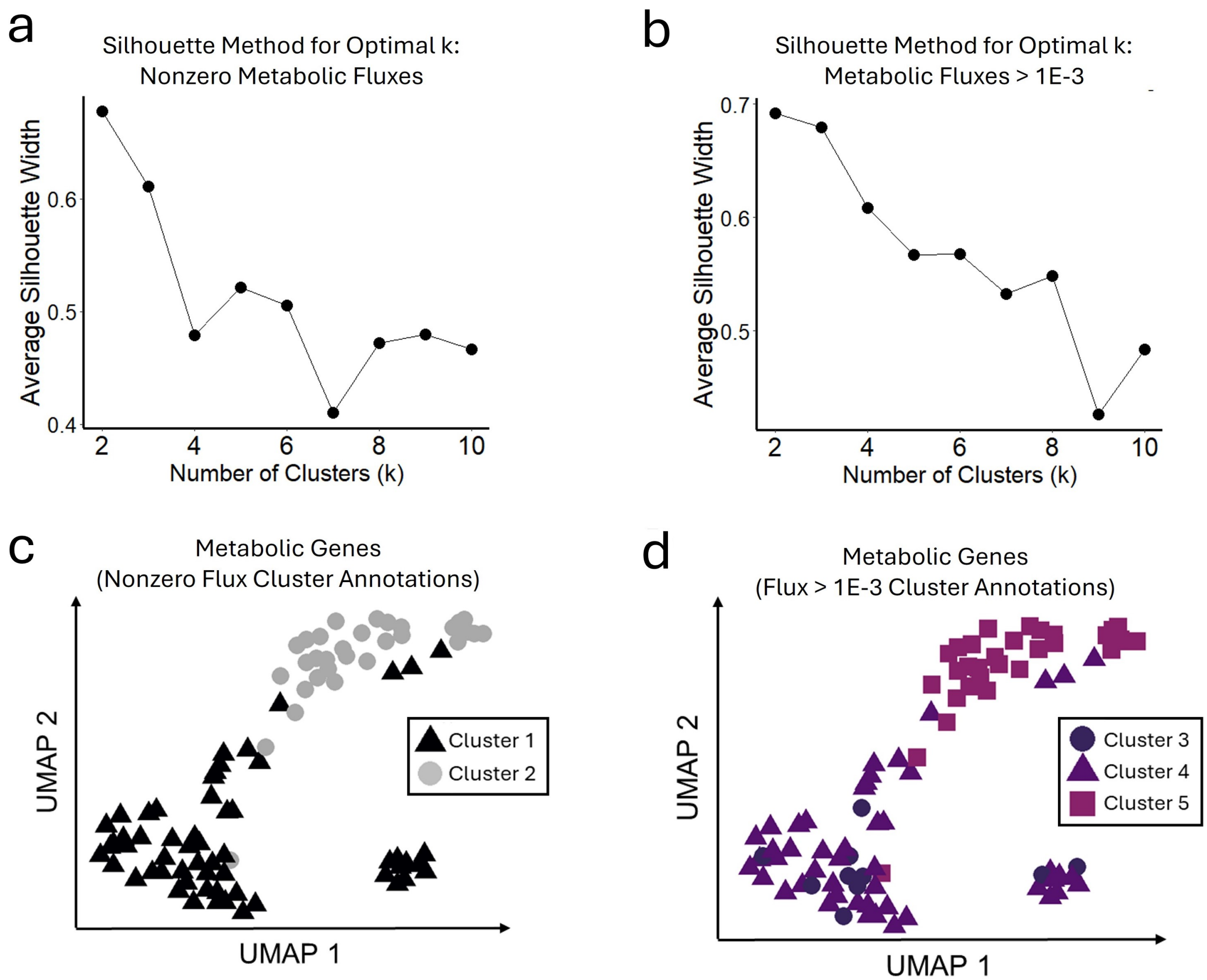


**Supplementary Fig. 2. Choosing optimal k for k-means clustering and overlaying metabolic flux annotations on metabolic gene expression UMAP.**
Silhouette analysis and plotting for optimal k in k-means clustering for (**a**) all nonzero metabolic fluxes and (**b**) metabolic fluxes > 0.001 mmol/gDW/g cutoff. Overlaying metabolic flux annotations on UMAP of metabolic genes alone for (**c**) Flux Cluster 1 and 2 (nonzero flux cluster annotations, resolution level 1) and (**d**) Flux Clusters 3, 4, and 5 (flux > 0.001 mmol/gDW/g cutoff cluster annotations, resolution level 2).

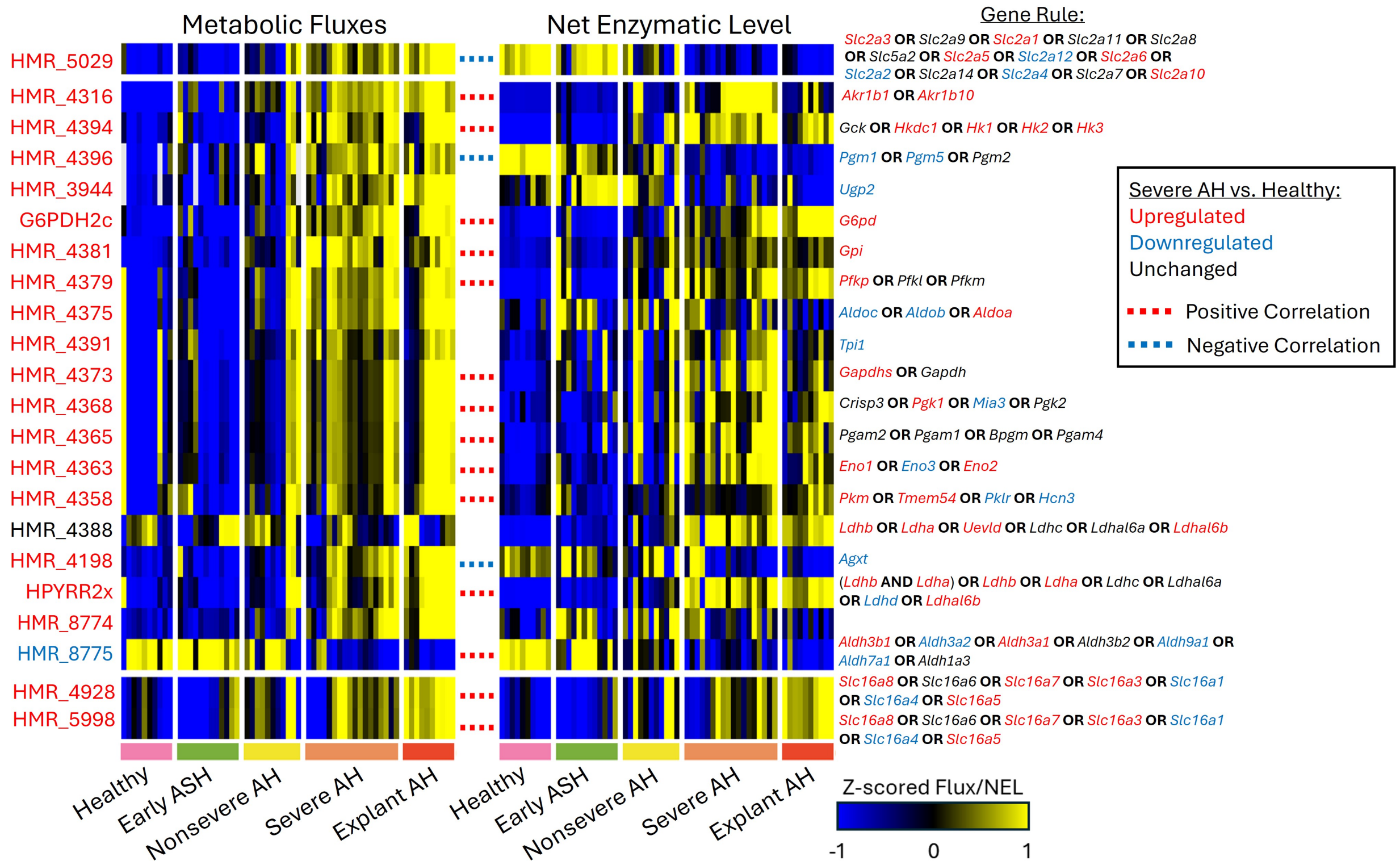


**Supplementary Fig. 3. Predicted metabolic fluxes and associated NELs for all reactions shown in Fig. 4a**.

Positive ($\rho > 0.5$, p-value $< 0.05$) and negative ($\rho < -0.5$, p-value $< 0.05$) Spearman correlations between the metabolic fluxes and NELs are shown in red and blue, respectively. In addition, the gene rules encoding each reaction are displayed with the genes colored by severe AH vs. healthy changes (upregulated = red, downregulated = blue, Unchanged = black).

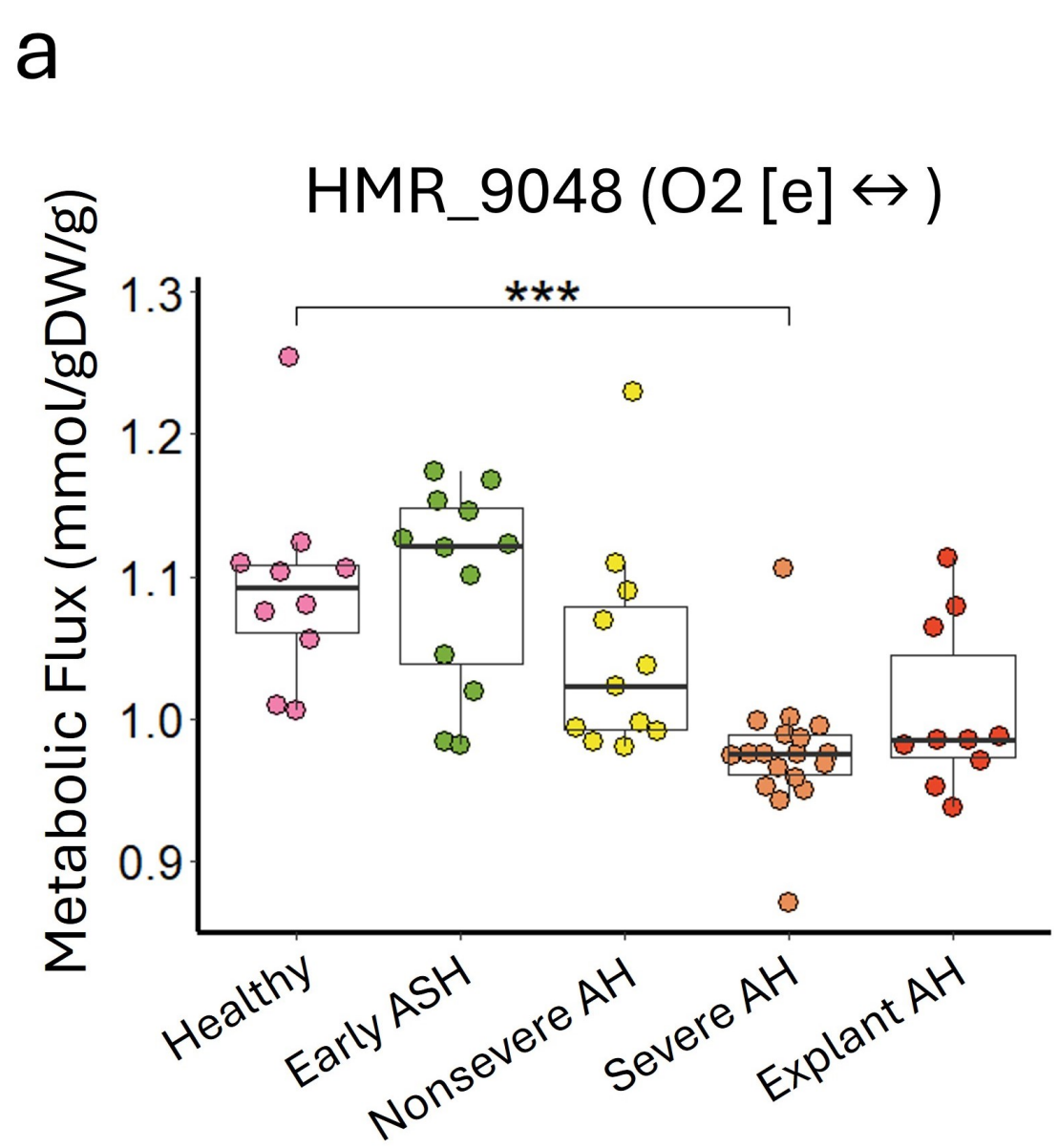


**Supplementary Fig. 4. Patients with AH have hypoxic liver conditions.**
Boxplots depicting the metabolic fluxes for (**a**) O2 exchange with the environment (HMR_9048: O2 [e] ↔ ) and (**b**) O2 import from the extracellular space to the cytosol (HMR_4896: O2 [e] → O2 [c]).

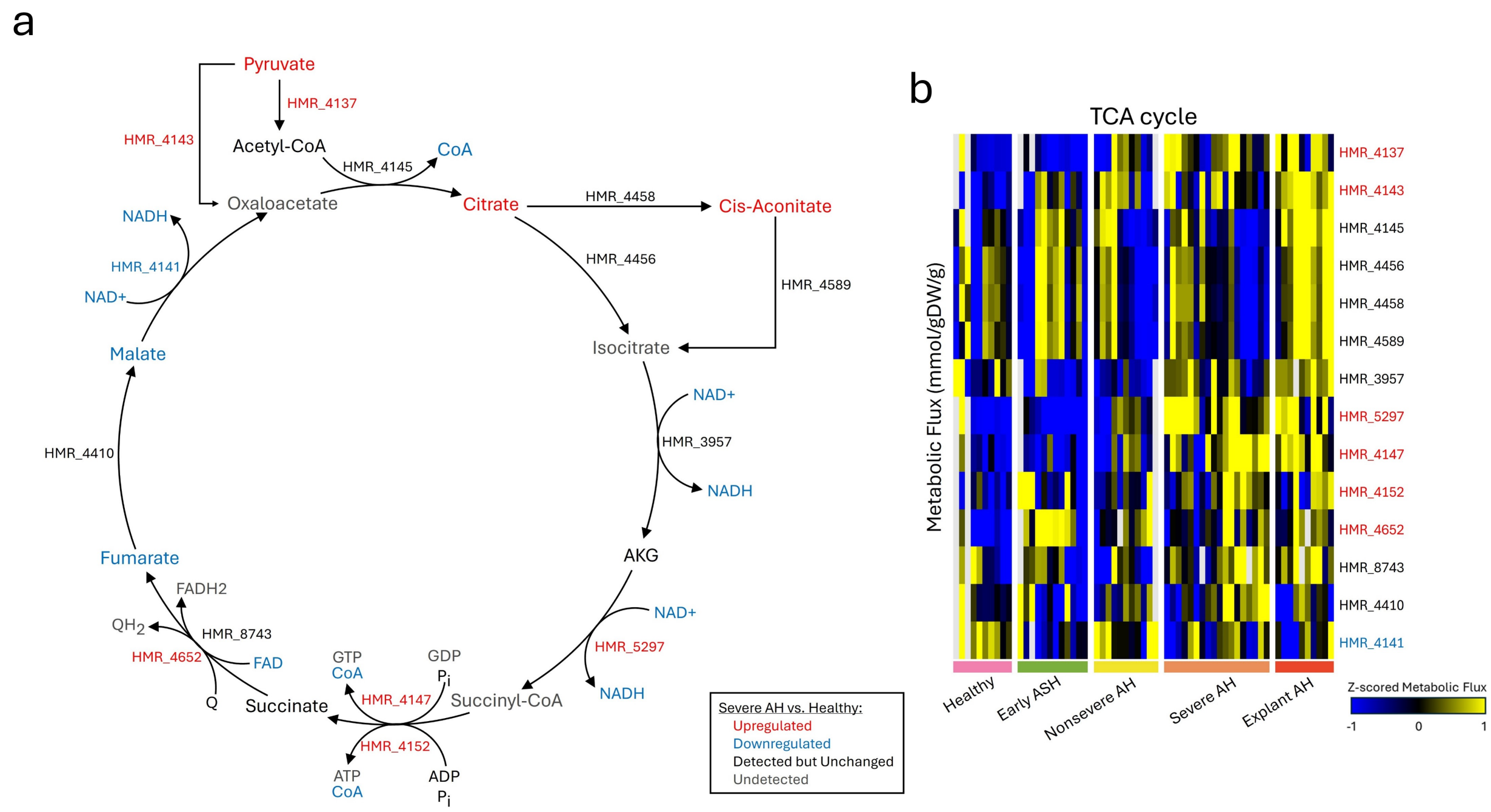


**Supplementary Fig. 5. TCA cycle metabolic map and fluxes.**
(**a**) Predicted fluxomes and publicly available metabolomics data [1] are overlaid on the TCA cycle metabolic map and show upregulated (red), downregulated (blue), detected but unchanged (black), and undetected (gray) levels for the severe AH vs. healthy control patient cohorts. (**b**) Z-scored TCA cycle fluxes are plotted in the form of a heatmap for healthy patients and those with ALD. Text color: Blue = downregulated, Red = upregulated, black = unchanged in severe AH vs. healthy control patients.

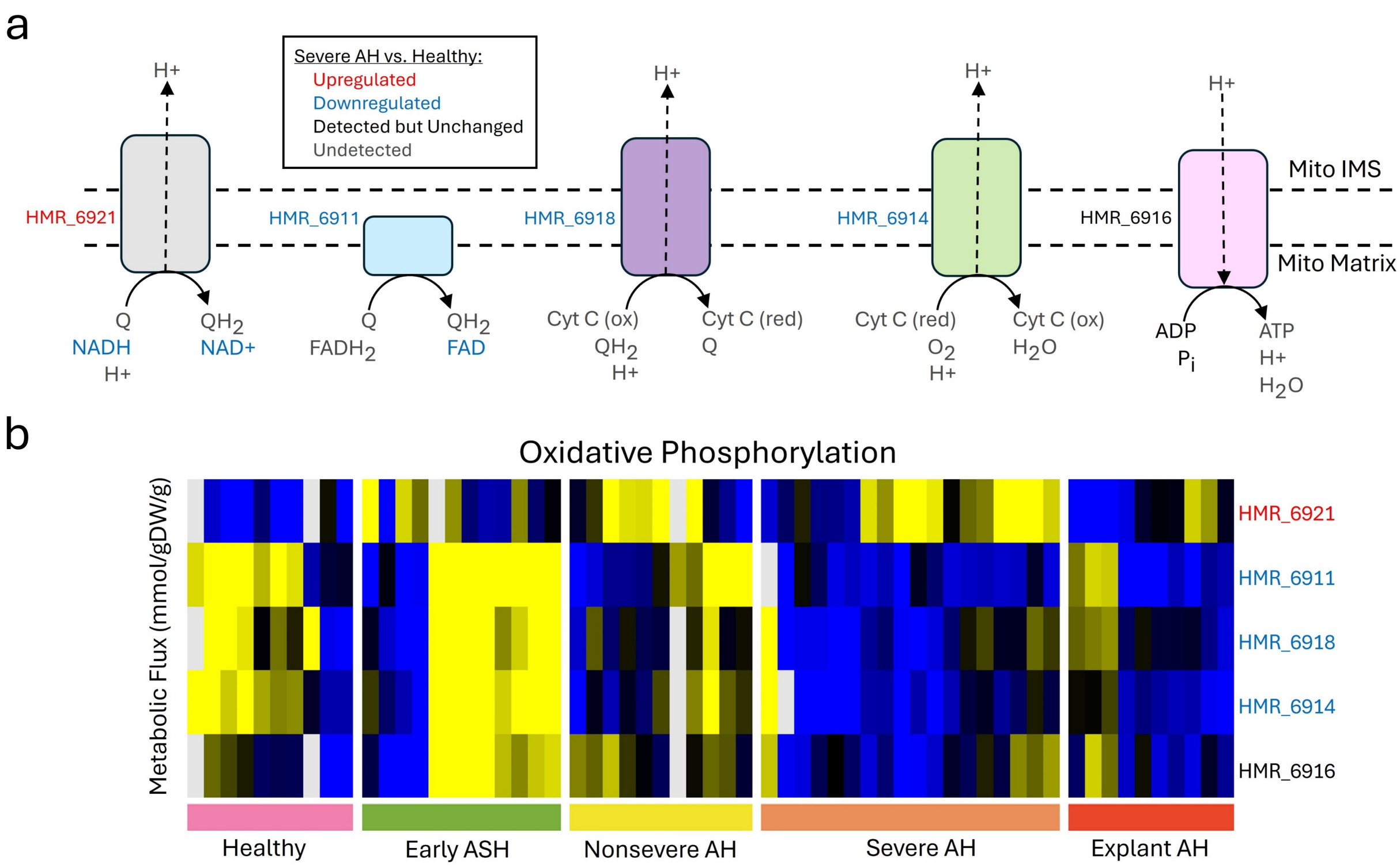


**Supplementary Fig. 6. Oxidative phosphorylation metabolic map and fluxes.**
(**a**) Predicted fluxomes and publicly available metabolomics data [1] are overlaid on the oxidative phosphorylation metabolic map and show upregulated (red), downregulated (blue), detected but unchanged (black), and undetected (gray) levels for the severe AH vs. healthy control patient cohorts. (**b**) Z-scored oxidative phosphorylation fluxes are plotted in the form of a heatmap for healthy patients and those with ALD. Text color: Blue = downregulated, Red = upregulated, black =unchanged in severe AH vs. healthy control patients. Mito IMS = mitochondrial intermembrane space, Mito Matrix = mitochondrial matrix. Q = ubiquinone, $QH_2$ = ubiquinol, Cyt C (red) = ferricytochrome C (reduced), Cyt C (ox): ferrocytochrome C (oxidized).

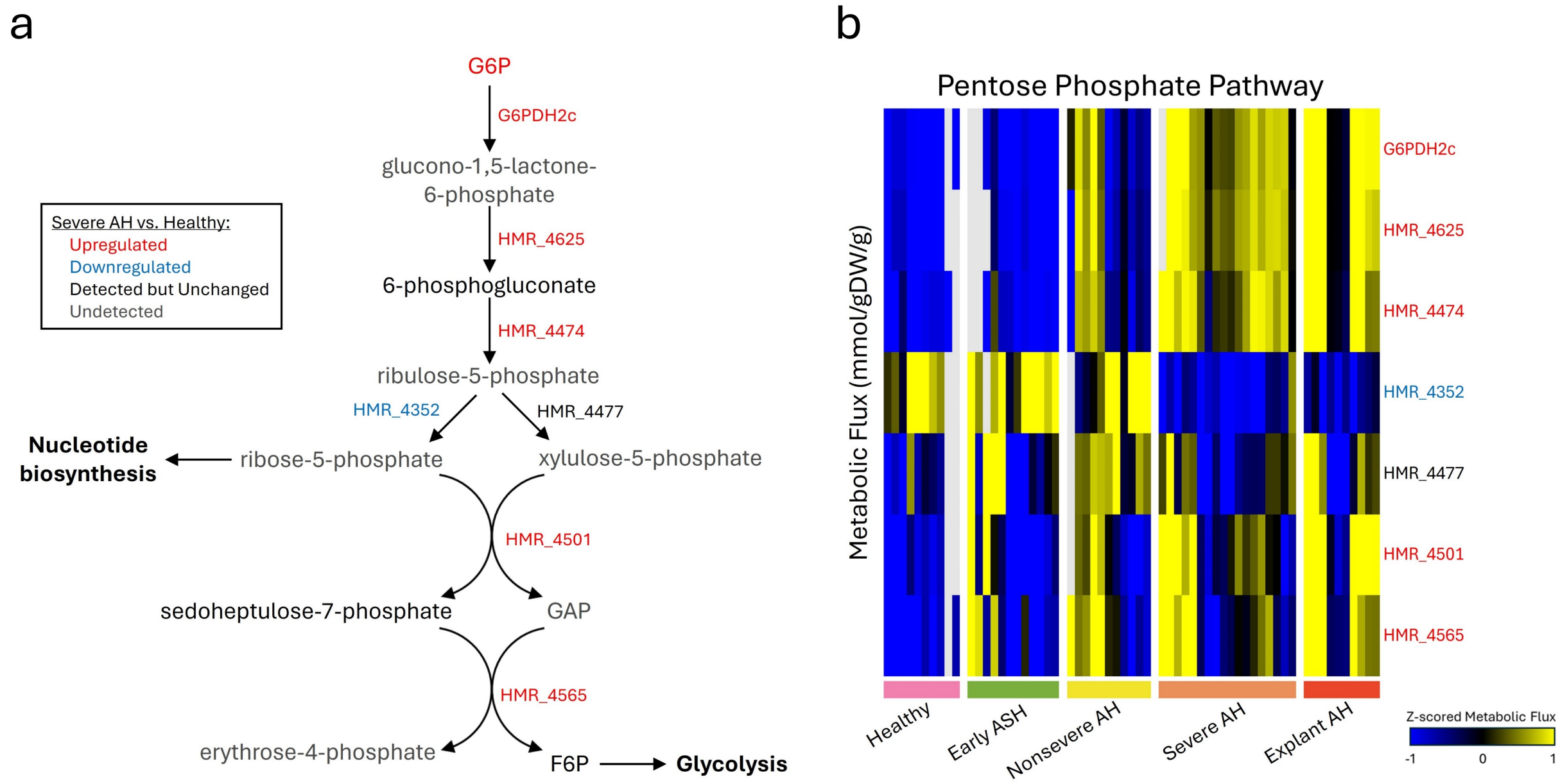


**Supplementary Fig. 7. Pentose phosphate pathway metabolic map and fluxes.**
(**a**) Predicted fluxomes and publicly available metabolomics data [1] are overlaid on the pentose phosphate pathway metabolic map and show upregulated (red), downregulated (blue), detected but unchanged (black), and undetected (gray) levels for the severe AH vs. healthy control patient cohorts. (**b**) Z-scored pentose phosphate pathway fluxes are plotted in the form of a heatmap for healthy patients and those with ALD. Text color: Blue = downregulated, Red = upregulated, black =unchanged in severe AH vs. healthy control patients.

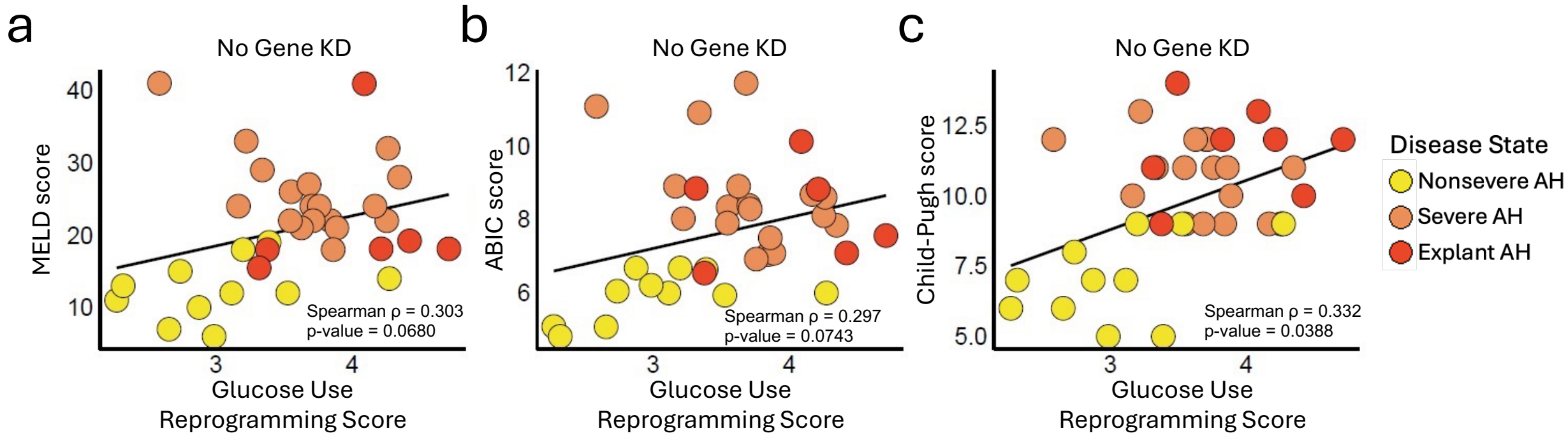


**Supplementary Fig. 8. Association between glucose use reprogramming scores and clinical scores of liver dysfunction in AH.**

Weak positive correlations for (**a**) MELD score, (**b**) ABIC scores, and (**c**) Child-Pugh scores vs. glucose use reprogramming scores.

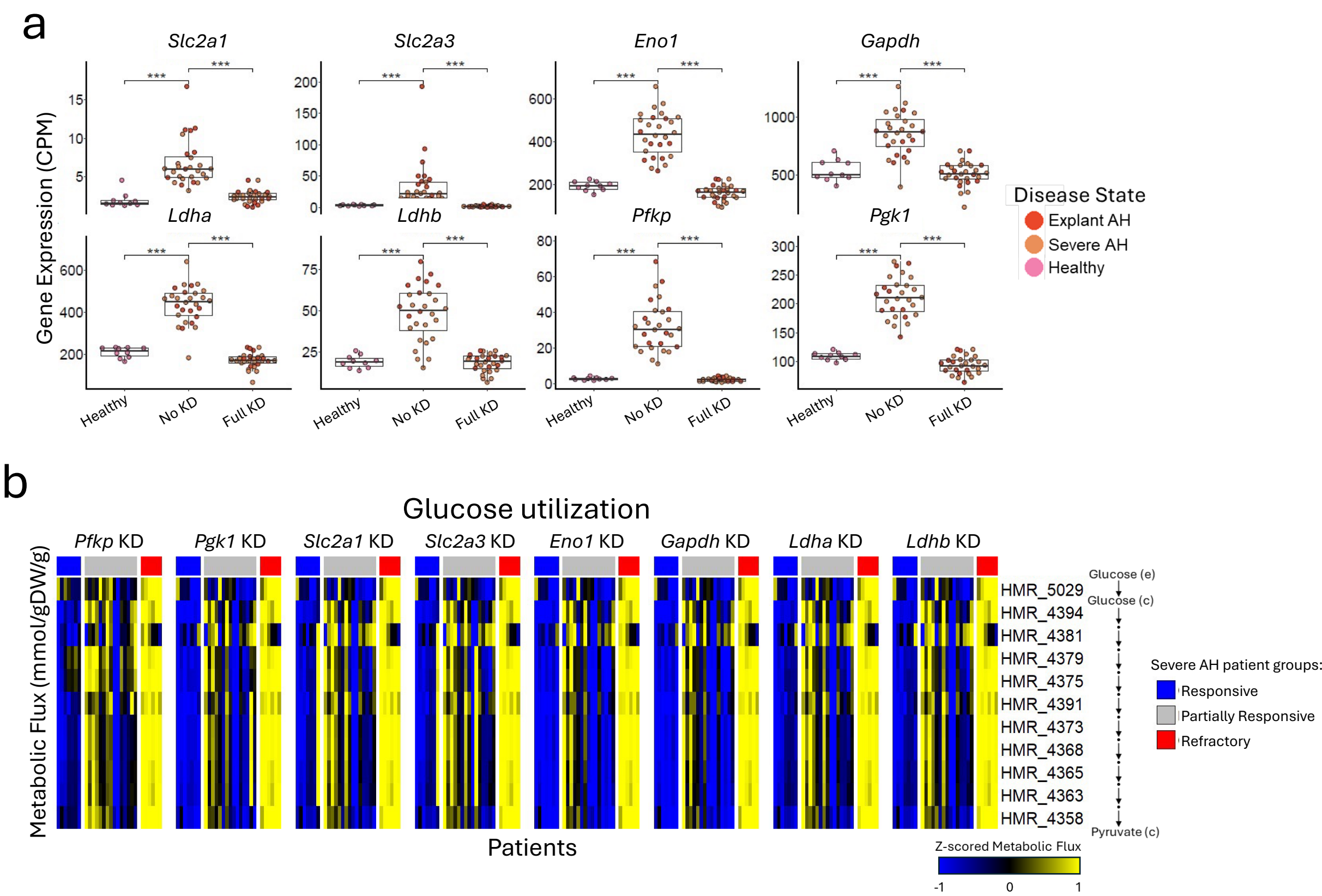


**Supplementary Fig. 9. *In silico* knockdown experiments that serve as a control for unchanging glucose utilization metabolic fluxes.**

(**a**) Knockdown of *Slc2a1, Slc2a3, Eno1, Gapdh, Ldha, Ldhb, Pfkp*, and *Pgk*, which are all genes in the glycolysis pathway that are upregulated with severe AH vs. healthy controls. (**b**) Following the knockdown of genes in S8A Fig, we identify no significant change in patient responses, with patients in the Refractory cohort and a subset of patients in the Partially Responsive cohort still exhibiting upregulated metabolic flux behavior in the glycolysis pathway. KD = knockdown. Significance testing: NS = not significant, * p-value < 0.05, ** p-value < 0.01, *** p-value < 0.001.

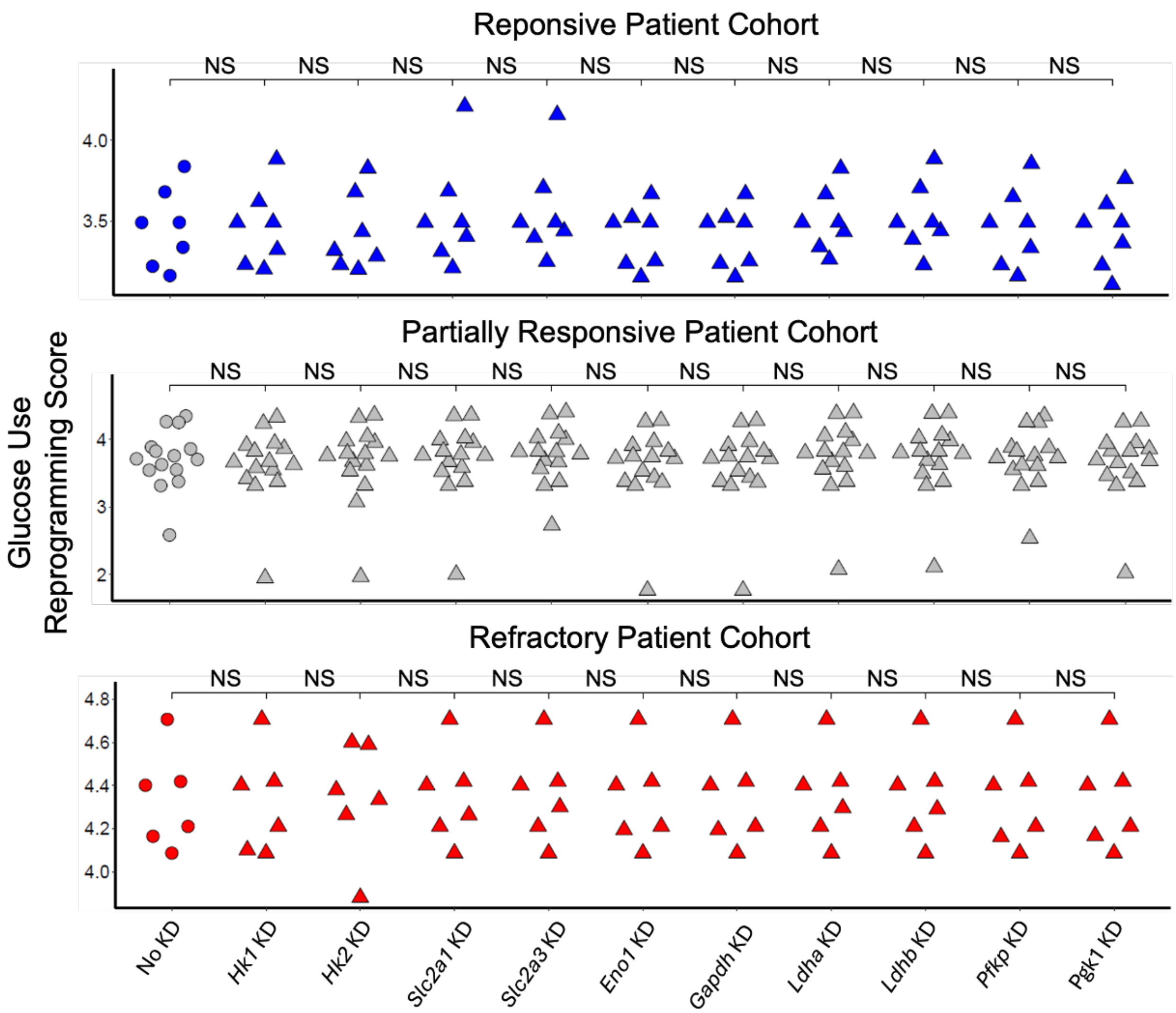


**Supplementary Fig. 10. Glucose use reprogramming scores for all control gene knockdown experiments.**

Individual knockdown of *Hk1, Hk2, Slc2a1, Slc2a3, Eno1, Gapdh, Ldha, Ldhb, Pfkp,* and *Pgk1* result in unchanging glucose use reprogramming scores for patients in the Responsive Patient Cohort (row 1), Partially Responsive Patient Cohort (row 2), or Refractory Patient Cohort (row 3). Significance testing: NS = not significant.

# References

1. Massey, V. *et al.* Integrated multiomics reveals glucose use reprogramming and identifies a novel hexokinase in alcoholic hepatitis. *Gastroenterology* **160**, 1725-1740.e2 (2021).
