# Supplementary material for: Patient-specific modeling identifies metabolic interventions for reversing glucose use reprogramming in alcohol-associated hepatitis
Source: Commun Biol. 2026 Jun 4;9:1181. doi: 10.1038/s42003-026-10407-5 (PMC13554195; doi:10.1038/s42003-026-10407-5)
Supplement: Supplementary file 3 [file 42003_2026_10407_MOESM3_ESM.pdf]

nature portfolio

Corresponding author(s): Rajanikanth Vadigepalli

Last updated by author(s): May 25, 2026

Reporting Summary

Nature Portfolio wishes to improve the reproducibility of the work that we publish. This form provides structure for consistency and transparency in reporting. For further information on Nature Portfolio policies, see our Editorial Policies and the Editorial Policy Checklist.

Statistics

For all statistical analyses, confirm that the following items are present in the figure legend, table legend, main text, or Methods section.

n/a	Confirmed	
☐	☒	The exact sample size (*n*) for each experimental group/condition, given as a discrete number and unit of measurement
☐	☒	A statement on whether measurements were taken from distinct samples or whether the same sample was measured repeatedly
☐	☒	The statistical test(s) used AND whether they are one- or two-sided<br>*Only common tests should be described solely by name; describe more complex techniques in the Methods section.*
☒	☐	A description of all covariates tested
☐	☒	A description of any assumptions or corrections, such as tests of normality and adjustment for multiple comparisons
☐	☒	A full description of the statistical parameters including central tendency (e.g. means) or other basic estimates (e.g. regression coefficient) AND variation (e.g. standard deviation) or associated estimates of uncertainty (e.g. confidence intervals)
☐	☒	For null hypothesis testing, the test statistic (e.g. *F*, *t*, *r*) with confidence intervals, effect sizes, degrees of freedom and *P* value noted<br>*Give P values as exact values whenever suitable.*
☒	☐	For Bayesian analysis, information on the choice of priors and Markov chain Monte Carlo settings
☒	☐	For hierarchical and complex designs, identification of the appropriate level for tests and full reporting of outcomes
☐	☒	Estimates of effect sizes (e.g. Cohen's *d*, Pearson's *r*), indicating how they were calculated

Our web collection on statistics for biologists contains articles on many of the points above.

Software and code

Policy information about availability of computer code

Data collection	The raw RNAseq data used in this study are openly available in the Database of Genotypes and Phenotypes (dbGAP) of the National Center for Biotechnology Information (United States National Library of Medicine, Bethesda, MD) under accession number phs001807.v1.p1.<br>In order to validate our method of glucose use reprogramming scoring, we utilized a publicly available, independent bulk RNAseq dataset consisting of 13 liver samples from patients with severe AH (GSE143318). All processed data files necessary for reproducing and replicating the modeling studies and figures are available via GitHub at https://github.com/Daniel-Baugh-Institute/Patient-specific_AH_LiverGEMStudy and via Zenodo (10.5281/zenodo.19472115).
Data analysis	The code for reproducing and replicating the modeling studies and figures are available via GitHub at https://github.com/Daniel-Baugh-Institute/Patient-specific_AH_LiverGEMStudy and via Zenodo (10.5281/zenodo.19472115).

For manuscripts utilizing custom algorithms or software that are central to the research but not yet described in published literature, software must be made available to editors and reviewers. We strongly encourage code deposition in a community repository (e.g. GitHub). See the Nature Portfolio guidelines for submitting code & software for further information.

Data

Policy information about availability of data

All manuscripts must include a data availability statement. This statement should provide the following information, where applicable:

- Accession codes, unique identifiers, or web links for publicly available datasets
- A description of any restrictions on data availability
- For clinical datasets or third party data, please ensure that the statement adheres to our policy

All data files necessary for reproducing and replicating the modeling studies and figures are available via GitHub at https://github.com/Daniel-Baugh-Institute/Patient-specific_AH_LiverGEMStudy and via Zenodo (10.5281/zenodo.19472115).

Research involving human participants, their data, or biological material

Policy information about studies with human participants or human data. See also policy information about sex, gender (identity/presentation), and sexual orientation and race, ethnicity and racism.

Reporting on sex and gender	We did not perform sex-based analysis as we did not have sufficient samples across sexes and hence focused on disease severity.
Reporting on race, ethnicity, or other socially relevant groupings	We do not have race, ethnicity, or other socially relevant grouping information available for this study.
Population characteristics	The 90 patient samples are stratified into seven distinct disease states and a healthy control state: explant alcoholic hepatitis (explant AH, n = 10), severe alcoholic hepatitis (severe AH, n = 18), non-severe alcoholic hepatitis (non-severe AH, n = 11), early alcoholic steatohepatitis (early ASH, n = 12), healthy (n = 10), compensated cirrhosis (comp. cirrhosis, n = 9), hepatitis C virus (HCV, n = 10), and non-alcoholic steatohepatitis (NASH, n = 9). Patients had a median age of 45 in the explant AH disease state category, 51 in severe AH, 46 in non-severe AH, 52 in early ASH, 32 in the healthy controls, 61 in compensated cirrhosis, 46 in HCV, and 49 in NASH.
Recruitment	The inclusion and exclusion criteria can be found at: https://www.ncbi.nlm.nih.gov/projects/gap/cgi-bin/study.cgi?study_id=phs001807.v1.p1
Ethics oversight	University of Pittsburgh

Note that full information on the approval of the study protocol must also be provided in the manuscript.

Field-specific reporting

Please select the one below that is the best fit for your research. If you are not sure, read the appropriate sections before making your selection.

☒ Life sciences ☐ Behavioural & social sciences ☐ Ecological, evolutionary & environmental sciences

For a reference copy of the document with all sections, see nature.com/documents/nr-reporting-summary-flat.pdf

Life sciences study design

All studies must disclose on these points even when the disclosure is negative.

Sample size	We used the available data from https://www.ncbi.nlm.nih.gov/projects/gap/cgi-bin/study.cgi?study_id=phs001807.v1.p1
Data exclusions	One explant AH patient sample was identified as an outlier (greater than six standard deviations above the mean) in the principal component analysis (PCA) plot and was removed from the downstream analysis.
Replication	In order to validate our method of glucose use reprogramming scoring, we utilized a publicly available, independent bulk RNAseq dataset consisting of 13 liver samples from patients with severe AH (GSE143318). The model predictions were reproducible across different data sets.
Randomization	We did not control for covariates. We used the available data from https://www.ncbi.nlm.nih.gov/projects/gap/cgi-bin/study.cgi?study_id=phs001807.v1.p1
Blinding	We did not participate in the group allocation in the data collection. We used the available data from https://www.ncbi.nlm.nih.gov/projects/gap/cgi-bin/study.cgi?study_id=phs001807.v1.p1

Reporting for specific materials, systems and methods

We require information from authors about some types of materials, experimental systems and methods used in many studies. Here, indicate whether each material, system or method listed is relevant to your study. If you are not sure if a list item applies to your research, read the appropriate section before selecting a response.

Materials & experimental systems

n/a	Involved in the study
☒	☐ Antibodies
☒	☐ Eukaryotic cell lines
☒	☐ Palaeontology and archaeology
☒	☐ Animals and other organisms
☐	☒ Clinical data
☒	☐ Dual use research of concern
☒	☐ Plants

Methods

n/a	Involved in the study
☒	☐ ChIP-seq
☒	☐ Flow cytometry
☒	☐ MRI-based neuroimaging

Clinical data

Policy information about clinical studies

All manuscripts should comply with the ICMJE guidelines for publication of clinical research and a completed CONSORT checklist must be included with all submissions.

Clinical trial registration: NCT02075918

Study protocol: https://clinicaltrials.gov/study/NCT02075918

Data collection: Study start: 05-2014. Study completion: 05-31-2019

Outcomes: Information on the outcome measure from https://clinicaltrials.gov/study/NCT02075918 is included below.

Primary outcome measure:
Diagnosis and Severity of Alcoholic Hepatitis
Utilizing liver tissue, blood samples, and stool and urine samples, InTeam will aim to discover and validate factors associated with the diagnosis and severity of Alcoholic hepatitis.

Secondary outcome measures:
Major Liver Complications - As an observational study, we will collect data regarding major liver complications such as, ascites, renal failure, encephalopathy, and bleeding.
Death/Transplantation - As an observational study, we will collect the data regarding number the survival of Alcoholic Hepatitis by considering deaths and transplantations.

Plants

Seed stocks: *Report on the source of all seed stocks or other plant material used. If applicable, state the seed stock centre and catalogue number. If plant specimens were collected from the field, describe the collection location, date and sampling procedures.*

Novel plant genotypes: *Describe the methods by which all novel plant genotypes were produced. This includes those generated by transgenic approaches, gene editing, chemical/radiation-based mutagenesis and hybridization. For transgenic lines, describe the transformation method, the number of independent lines analyzed and the generation upon which experiments were performed. For gene-edited lines, describe the editor used, the endogenous sequence targeted for editing, the targeting guide RNA sequence (if applicable) and how the editor was applied.*

Authentication: *Describe any authentication procedures for each seed stock used or novel genotype generated. Describe any experiments used to assess the effect of a mutation and, where applicable, how potential secondary effects (e.g. second site T-DNA insertions, mosiacism, off-target gene editing) were examined.*
